# Nitrosyl and Thionitrosyl Complexes of Technetium and Rhenium and Their Reactions with Hydrotris(pyrazolyl)borates

**DOI:** 10.3390/molecules29163865

**Published:** 2024-08-15

**Authors:** Till Erik Sawallisch, Abdullah Abdulkader, Domenik Nowak, Adelheid Hagenbach, Ulrich Abram

**Affiliations:** Institute of Chemistry and Biochemistry, Freie Universität Berlin, Fabeckstr. 34/36, 14195 Berlin, Germany

**Keywords:** technetium, rhenium, nitrosyl complexes, thionitrosyl complexes, pyrazolylborates

## Abstract

The very limited number of structurally known thionitrosyl complexes of technetium was increased by the synthesis of [Tc^II^(NS)Cl_3_(PPh_3_)_2_] (**3**) and [Tc^II^(NS)Cl_3_(PPh_3_)(OPPh_3_)] (**4**) and their reaction products with hydrotris(pyrazolyl)borates, {HB(pz^R^)_3_}^−^. Similar reactions were conducted with [Tc^I^(NO)Cl_2_(PPh_3_)_2_(CH_3_CN)] and related rhenium thionitrosyls. Remarkably, most such reactions result in a rapid cleavage of the boron–nitrogen bonds of the ligands and the formation of pyrazole complexes of the two group 7 metals. Only one compound with an intact {HB(pz^R^)_3_}^−^ ligand could be isolated: the technetium(I) complex [Tc^I^(NO)Cl(PPh_3_){HB(pz)_3_}] (**2**). Other products show the coordination of one or four neutral pyrazole ligand(s) in the coordination spheres of technetium generated by thermal decomposition of the pyrazolylborates [Tc^I^(NO)Cl_2_(PPh_3_)_2_(pz^H^)] (**1**) and [Tc^I^(NS)Cl(pz^HMe2^)_4_]^+^ (**5**). Reactions with the corresponding thionitrosylrhenium complex [Re^II^(NS)Cl_3_(PPh_3_)_2_] require higher temperatures and only compounds with one pyrazole ligand, [Re^I^(NS)Cl_2_(PPh_3_)(pz^HR^)] (**6a**–**6c**), were isolated. The products were studied spectroscopically and by X-ray diffraction.

## 1. Introduction

**Figure 1:**
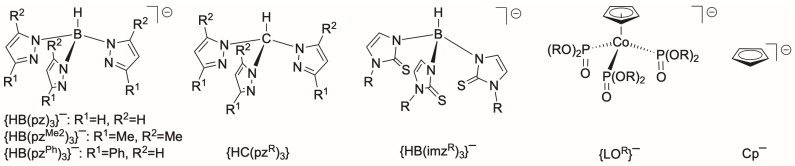
Hydrotris(pyrazolyl)borates, {HB(pz^R^)_3_}^−^, used in the present paper and related isolobal ligands.

Hydrotris(pyrazolyl)borates, {HB(pz^R^)_3_}^−^, belong to the remarkable family of scorpionates, where these tripodal ligands are accompanied by tris(pyrazolyl)methanes, {HC(pz^R^)_3_}, hydrotris(mercaptoimidazolyl)borates, {HB(imz^R^)_3_}^−^, (η^5^-cyclopentadienyl)tris(dialkyl phosphito-*P*)cobaltates(III), {L^OR^}^−^ (Figure 1), and related ligand systems such as nitrogen crown ethers or thiacrown ethers. Such compounds commonly coordinate metal ions in a facial manner. They are isolobal with Cp^−^ ligands and have found wide recognition in various fields of coordination chemistry [1,2,3,4,5,6,7,8]. Technetium complexes with {HB(pz^R^)_3_}^−^ ligands are rare and mainly concern trioxido complexes of technetium(VII) or carbonyltechnetium(I) compounds [9,10,11,12,13], while the coordination chemistry of rhenium with scorpionates is more diverse [8,10,14,15,16,17,18,19]. There are also reports about potential applications of scorpionate complexes of technetium and rhenium complexes in nuclear medical procedures on the basis of tricarbonyl compounds [19,20,21,22]. Comparable compounds with nitrosyl or thionitrosyl units are not yet known.

In general, the chemistry of nitrosyls of the radioactive element technetium [23,24,25,26,27,28,29,30,31,32,33,34,35,36,37,38,39,40,41,42,43,44,45] is not as thoroughly described as that of the stable rhenium [25,46,47,48,49,50,51,52,53]. Less is known about the corresponding thionitrosyl complexes of both elements [54,55,56,57,58,59,60,61,62,63,64,65,66,67,68,69,70,71,72,73]. This is most probably due to the lack of a readily available monomeric nitrogen sulfide. Although some NS^+^ salts have been isolated [74,75,76,77], their synthesis is still a challenge. Only occasionally have they been used as starting materials for the synthesis of thionitrosyl complexes [54,76,77]. Other suitable routes for the formation of NS^+^ or NSCl^+^ ligands have been established starting from cyclic tetrasulfur tetranitride, S_4_N_4_, or trithiazyltrichloride, (NSCl)_3_. Such reactions, however, sometimes give unpredictable products. A more or less convenient access to low-valent thionitrosyl compounds, however, is given by the addition of sulfur atoms to nitrido ligands whenever corresponding high-valent nitrido complexes are available. Such reactions proceed with a variety of sulfur sources, but the use of S_2_Cl_2_ usually gives the best and most reproduceable results. It has also been used for the synthesis of some rhenium and technetium thionitrosyls with the metals in their “+1” to “+3” oxidation states [66,67,69,70,71,73,78].

## 2. Results and Discussion

### 2.1. Nitrosyl Complexes of Technetium

**Scheme 1:**
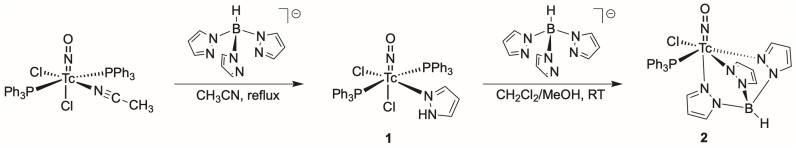
Subsequent reactions of [Tc(NO)Cl_2_(PPh_3_)_2_(CH_3_CN)] and [Tc(NO)Cl_2_(PPh_3_)_2_(pz^H^)] with K{HB(pz)_3_).

Recently, the syntheses of stable nitrosyltechnetium complexes with some of the tripodal ligands shown in Figure 1 were reported [30,43], and in particular, the Cp^−^ derivative [Tc(NO)Cl(Cp)(PPh_3_)] shows an extended ligand exchange chemistry. During such reactions, the robust {Tc(NO)(Cp)(PPh_3_)}^+^ core is essentially retained, while a variety of interesting reactions were observed at the vacant sixth coordination position [30,31,32,79]. Products of similar stability with a “fixed” {Tc(NO)(PPh_3_)(L^OMe^)}^+,2+^ core were obtained when common nitrosyltechnetium complexes such as [Tc^I^(NO)Cl_2_(PPh_3_)(CH_3_CN)] or [Tc^II^(NO)Cl_4_(MeOH)]^−^ were exposed to the “Kläui-type” ligand {L^OMe^}^−^ [39,43]. The ready availability of the mentioned scorpionate complexes stimulated us to perform similar reactions with the classical Trofimenko ligand {HB(pz)_3_}^−^ (Scheme 1). 

**Figure 2:**
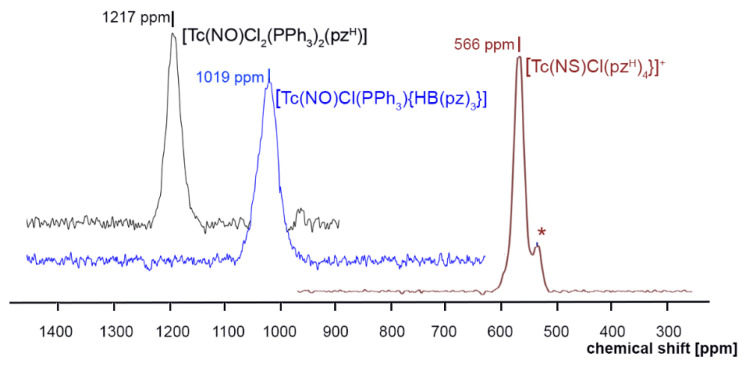
^99^Tc NMR spectra of [Tc(NO)Cl_2_(PPh_3_)_2_(pz^H^)] (**1**), [Tc(NO)Cl(PPh_3_){HB(pz)_3_}] (**2**) and [Tc(NS)Cl(pz^H^)_4_]Cl (**5**Cl) (* indicates a small amount of [Tc(NS)(pz^H^)_4_]^2+^).

The starting material [Tc(NO)Cl_2_(PPh_3_)(CH_3_CN)] is only sparingly soluble, but subsequently dissolves during ligand exchange procedures under formation of the products. Such reactions are commonly performed under elevated temperatures to increase the reaction rate. Heating was also required for the attempted reaction of [Tc^I^(NO)Cl_2_(PPh_3_)(CH_3_CN)] with K{HB(pz^H^)_3_}, since at room temperature no consumption of the starting complex was observed irrespective of the solvents used. Upon heating in acetonitrile, however, a red solution was rapidly formed and a non-radioactive, colorless solid precipitated. After filtration, a red solid could be isolated (compound **1**), the IR spectrum of which showed a ν_NO_ band at 1708 cm^−1^. The frequency range of this absorption is a clear indicator of the formation of a nitrosyltechnetium(I) complex, since the ν_NO_ signals of Tc(II) complexes appear at markedly higher wavenumbers due to the lower extent of back-donation in anti-bonding MOs of the nitrosyl ligands in such compounds. The diamagnetism of the product is confirmed by the detection of resolved ^1^H NMR spectra with narrow lines and the detection of a ^99^Tc NMR signal at 1217 ppm relative to TcO_4_^−^ (Figure 2). This chemical shift is within the range that has been previously found for nitrosyltechnetium(I) compounds [27]. An analysis of the ^1^H spectrum indicates that it was not a simple ligand exchange reaction with the insertion of an intact {HB(pz)_3_}^−^ ligand that took place. This assumption was confirmed by the results of an X-ray diffraction study (vide infra). The ^31^P NMR spectrum of the product is characterized by a very broad line at about 35 ppm, which is accompanied by a narrow signal at 20 ppm (most probably a minor impurity of OPPh_3_). The broadening of ^31^P NMR lines is frequently observed in phosphine complexes of technetium when the local symmetry around the metal atom is low. A common explanation is given by scalar couplings with the large quadrupole moment of ^99^Tc (Q = –0.19 Å·10^−28^ m^2^) [80,81]. Such interactions can cause extreme line-broadenings, which frequently make the resolution of ^31^P signals impossible [82,83,84].

**Figure 3:**
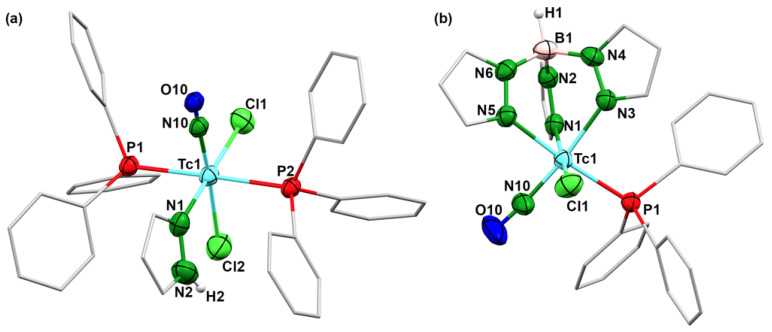
Molecular structures of (**a**) [Tc(NO)Cl_2_(PPh_3_)_2_(pz^H^)] (**1**) and (**b**) [Tc(NO)Cl(PPh_3_){HB(pz)_3_}] (**2**). Thermal ellipsoids show 50 per cent probability.

Red single crystals of compound **1** were obtained from a CH_2_Cl_2_/MeOH mixture. A representation of the molecular structure is shown in Figure 3a. It clearly confirms the information derived from the NMR spectra: that the potentially tripodal hydrotris(pyrazolyl)borate ligand decomposed under the elevated temperatures used during the reaction and/or under the influence of the transition metal. The product contains only one of the formed pyrazole molecules, which is coordinated in the equatorial coordination sphere of the resulting complex [Tc(NO)Cl_2_(PPh_3_)_2_(pz^H^)] (**1**) in trans position to a chlorido ligand. Linear coordination is observed for the nitrosyl ligand, which is in agreement with all hitherto structurally studied NO complexes of technetium and supports treatment as a NO^+^ unit. Remarkably, the Tc–Cl bonds have almost the same lengths, which suggests that the structural trans influences induced by NO^+^ and pyrazole ligands are similar.

Cleavage of the boron–nitrogen bonds in hydrotris(pyrazolyl)borates and related compounds is not without precedence. It has also been observed during reactions with other metal ions, and the released pyrazoles are commonly found as ligands in the products [85,86,87,88,89]. Such ligand decompositions are frequently observed at elevated temperatures, as in the attempted reaction with [Tc(NO)Cl_2_(PPh_3_)_2_(CH_3_CN)]. Since all attempts at reactions of K{HB(pz)_3_} with this almost insoluble starting material at room temperature failed, we used the newly prepared pyrazol complex **1** as a precursor. [Tc(NO)Cl_2_(PPh_3_)_2_(pz^H^)] (**1**) readily dissolves in a CH_2_Cl_2_/MeOH mixture and reacts at room temperature with K{HB(pz)_3_} causing formation of the desired scorpionate complex [Tc(NO)Cl(PPh_3_){HB(pz)_3_}] (**2**). Compound **2** precipitated as an orange-brown solid directly from the reaction mixture. Single crystals of the product suitable for X-ray diffraction were grown from the same solvents.

The structural analysis of **2** confirmed that an intact scorpionate ligand is coordinated to technetium in the expected tripodal mode (Figure 3b). Selected bond lengths and angles are given in Table 1 and compared with corresponding values in the pyrazole complex **1**. In the IR spectrum of **2**, the ν_NO_ stretch appears at 1718 cm^−1^, which is close to the value obtained for **1** and clearly confirms the “+1” oxidation state of technetium in the product. The diamagnetism of the d^6^ system allows the recording of NMR spectra. Figure 3 contains the ^99^Tc spectrum at 1019 ppm, which is close to the signal of the nitrosyl complex **1**. Both signals are relatively broad with line-widths between 3000 and 4000 Hz. Such line-widths, however, are not unusual for technetium complexes with low local symmetry and are explained by the large quadruole moment of ^99^Tc [80]. Extremely narrow signals are usually only obtained for corresponding compounds with highly symmetric coordination spheres, such as in octahedral [Tc^I^(L)_6_]^+^ cations or in the tetrahedral Tc^VII^O_4_^−^ anion [81].

### 2.2. Thionitrosyl Complexes of Technetium

**Scheme 2:**
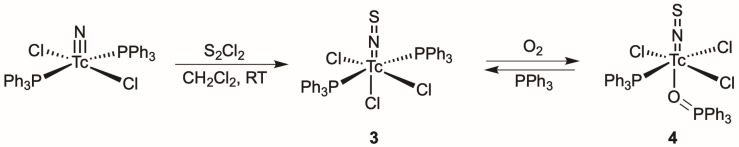
Synthesis of thionitrosyltechnetium(II) complexes with PPh_3_ ligands.

In contrast to the, to some extent, well-known nitrosyl chemistry of technetium [23,24,25,26,27,28,29,30,31,32,33,34,35,36,37,38,39,40,41,42,43,44,45], thionitrosyl compounds of this element are rare. The general synthetic route for the preparation of thionitrosyls, the addition of sulfur atoms to coordinated nitrido ligands, also works for technetium. S_2_Cl_2_ is a suitable sulfur source for this approach, but reactions with other sulfur-containing compounds such as SOCl_2_ [66], dithionite [72] or SCN^−^ [62] have also been reported for the (mostly unintended) synthesis of Tc thionitrosyls. Reactions of (NBu_4_)_2_[TcCl_6_] with (NSCl)_3_ give the Tc(II) anion [Tc(NS)Cl_4_]^−^ in good yields. The product, however, is not stable in solution and gradually decomposes leading to the formation of [Tc^VI^NCl_4_]^−^ and elemental sulfur [73]. Since nitridotechnetium complexes with {HB(pz)_3_}^−^ ligands were not available, we decided to prepare PPh_3_ complexes as starting materials. Corresponding procedures for the rhenium analogs have recently been reported [78], and the general synthetic route can also be applied for technetium (Scheme 2).

[TcNCl_2_(PPh_3_)_2_] is a sparingly soluble technetium(V) complex that found widespread application in the synthesis of other nitrido compounds [90,91]. Reactions of this complex with an excess of disulfur dichloride in CH_2_Cl_2_ at room temperature proceed with a rapid dissolution of the starting material and the formation of a green solution, when performed in an inert atmosphere. Similar observations can be made during corresponding reactions in air: the nitrido complex readily dissolves and an initially green solution is formed, indicating the formation of [Tc(NS)Cl_3_(PPh_3_)_2_] (**3**). Under aerobic conditions, however, the reaction does not stop at this stage, but proceeds with the gradual formation of the red oxidation product [Tc(NS)Cl_3_(PPh_3_)(OPPh_3_)] (**4**). As a solid, complex **3** is stable and can be stored for several months. Solutions of compound **3**, however, are sensitive to oxidation and the mixed phosphine/phosphine oxide complex **4** can also be prepared from isolated [Tc(NS)Cl_3_(PPh_3_)_2_] (**3**). Conversely, the addition of PPh_3_ to solutions of **4** and heating re-forms compound **3** in good yields.

**Figure 4:**
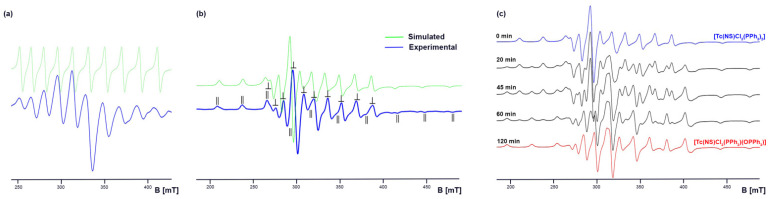
Solution EPR spectra of [Tc(NS)Cl_3_(PPh_3_)_2_] (**3**) in CH_2_Cl_2_ (**a**) at room temperature and (**b**) at T = 77 K (assignment of the ^99^Tc lines to the parallel (‖) and perpendicular (⊥) parts of the spectrum is indicated). (**c**) Frozen solution EPR spectra of a reaction sequence of **3** in CH_2_Cl_2_ under aerobic conditions, indicating the gradual conversion of [Tc(NS)Cl_3_(PPh_3_)_2_] into [Tc(NS)Cl_3_(PPh_3_)(OPPh_3_)].

The course of such reactions can be estimated by the color of the reaction mixtures (green to red or reverse). A more indicative monitor is the measurement of successive EPR spectra of the reaction mixtures. As 4d^5^ “low-spin” complexes, compounds **3** and **4** are S = ½ systems with one unpaired electron. This allows the measurement of resolved solution EPR spectra. Representative spectra of [Tc(NS)Cl_3_(PPh_3_)_2_] are depicted in Figure 4a,b together with their simulations. A frozen-solution spectrum of compound **4** is shown as the final product of the reaction sequence in Figure 4c. 

Interactions of the unpaired electron with the nuclear spin of ^99^Tc (I = 9/2) result in the formation of ten-line patterns in the room temperature spectra (Figure 4a), while the spectra of frozen solutions indicate essentially axial symmetry with well-resolved ^99^Tc hyperfine lines in the parallel and perpendicular parts. The assignment of the individual lines is indicated in Figure 4b. In contrast to the spectra of corresponding rhenium complexes [60,78], a marked g value anisotropy is observed and no significant rhombic distortion of the tensor components is detected. This may be attributed to the significantly different spin orbit coupling constants of the respective central ions ($\lambda_{{Tc}^{2+}}=950$ cm^−1^, $\lambda_{{Re}^{2+}}=2100$ cm^−1^) [92]. Couplings due to interactions with the ^31^P nuclei of the coordinated phosphines are not resolved for the PPh_3_ complexes in this study. Interestingly, corresponding line-splittings are characteristic of the parallel part lines of the frozen-solution spectra of similar nitrosyl- and thionitrosyltechnetium(II) complexes with dimethylphenylphosphine (PMe_2_Ph) ligands, where they cause doublet or triplet structures [67,70,93]. The absence of such splitting in the EPR spectra of **3** and **4** may be caused by the somewhat broader ^99^Tc hyperfine lines, but may also due to a lower extent of delocalization of the unpaired electron into ligand orbitals of PPh_3_, which is indicated by the clearly larger ^99^Tc hyperfine couplings A^Tc^ for **3** and **4** compared with the values obtained for corresponding PMe_2_Ph complexes. Table 2 contains a summary of the EPR parameters derived for the thionitrosyl complexes of the present study, together with the values for [Tc(NS)Cl_3_(PMe_2_Ph)_2_], [Tc(NS)Cl_3_(PMe_2_Ph)(OPMe_2_Ph)] and [Tc(NO)Cl_3_(PMe_2_Ph)_2_].

The differences in the g values and ^99^Tc coupling constants between [Tc(NS)Cl_3_(PPh_3_)_2_] and [Tc(NS)Cl_3_(PPh_3_)(OPPh_3_)] allow for ready differentiation of signals of the two compounds in the respective spectra, particularly in their parallel parts, and qualitative (and even semiquantitative) estimations do not require full spectral analyses. Thus, the course of the gradual oxidation of one of the PPh_3_ ligands of **3**, which is accompanied by the conversion of **3** into **4,** can be readily estimated on the basis of the clearly separated low-field lines of the parallel parts of the spectra. This is illustrated in Figure 4c. The presence of the EPR signals of (only) two different paramagnetic compounds (**3** and **4**) in the course of the reaction is apparent, and after two hours practically all **3** is consumed during formation of **4**.

**Figure 5:**
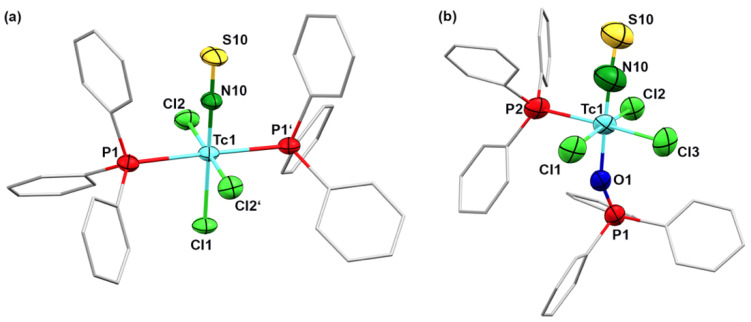
Molecular structures of (**a**) [Tc(NS)Cl_3_(PPh_3_)_2_] (**3**) and (**b**) [Tc(NS)Cl_3_(PPh_3_)(OPPh_3_)] (**4**). Thermal ellipsoids show 50 per cent probability.

The spectral parameters discussed above reflect the structural changes that are related to the oxidation of one PPh_3_ ligand. The newly formed OPP_3_ ligand is directed into trans position of the NS^+^ ligand, while the two PPh_3_ ligands in **3** are coordinated trans to each other in the equatorial coordination sphere. Such behavior is not unusual for complexes with multiply bonded ligands, where the ligands with ‘hard’ donor atoms are arranged in the trans position of, e.g., metal–nitrogen multiple bonds [25,29,35,43,49,67]. The structures of the two complexes are shown in Figure 5 and selected bond lengths and angles are summarized in Table 3. 

Having in mind the results of the reaction of the nitrosyl complex [Tc(NO)Cl_2_(PPh_3_)_2_(CH_3_CN)] with hydrotris(pyrazolyl)borate, which proceeded with B–N bond cleavages and the formation of a product with one pyrazole ligand (Scheme 1), it appeared interesting to perform a similar reaction with the related thionitrosyl complex **3** as well. Unlike the nitrosyl starting material, **3** is sufficiently soluble in common solvents and, thus, the reaction can be performed at room temperature. A potential thermal decomposition of the ligand should be avoided in this way.

**Scheme 3:**
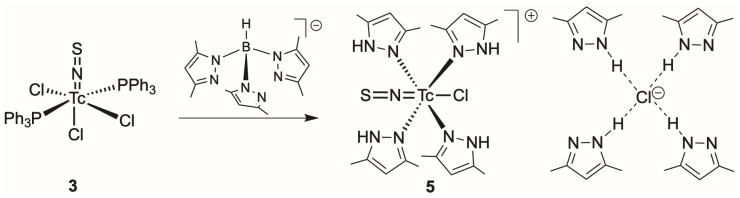
Reaction of [Tc(NS)Cl_3_(PPh_3_)_2_] with K{HB(pz^Me2^)_3_}.

Treatment of a solution of [Tc(NS)Cl_3_(PPh_3_)_2_] in CH_2_Cl_2_ with a methanolic solution containing an excess of K{HB(pz^Me2^)_3_} resulted in an immediate color change from red to brown. From this solution, the unusual product [Tc(NS)Cl(pz^HMe2^)_4_]{Cl(pz^HMe2^)_4_} (**5**{Cl(pz^HMe2^)_4_}) could be isolated; see Scheme 3. An EPR and ^99^Tc NMR monitoring of the reaction mixture confirms rapid consumption of the paramagnetic starting material, while several NMR signals appeared. After prolonged stirring at room temperature, a ^99^Tc NMR signal at 566 ppm dominated. It belongs to cation **5,** as could be confirmed by a single crystal X-ray analysis of the pale blue crystals isolated from the reaction mixture in moderate yields. Solutions of these crystals show the same ^99^Tc chemical shift together with a minor amount of a second compound in the same spectral region (see Figure 2c), which indicates some dynamic processes in the solution. The second species might be assigned to [Tc(NS)(pz^HMe2^)_4_]^2+^ or [Tc(NS)(OPPh_3_)(pz^HMe2^)_4_]^2+^ cations, since one equivalent of co-crystallized triphenylphosphine oxide was found in the respective crystals. The downfield shift of the signal of the thionitrosyl complex, compared with those of the nitrosyl compounds shown in Figure 2, is remarkable. It is, to the best of our knowledge, only the second ^99^Tc chemical shift reported for a technetium thionitrosyl complex and it appears close to the signal found for the only other compound: [Tc(NS)Cl_2_(PMe_2_Ph)_3_] (645 ppm) [71]. The difference of several hundred ppm between the values found for the nitrosyl and thionitrosyl complexes of the present study should not be overestimated, given the fact that the ^99^Tc chemical shifts observed for different nitrosyltechnetium(I) complexes also span a range of almost 3500 ppm from +2000 to −1500 ppm, depending on the π-acceptor properties of the other ligands [27].

**Figure 6:**
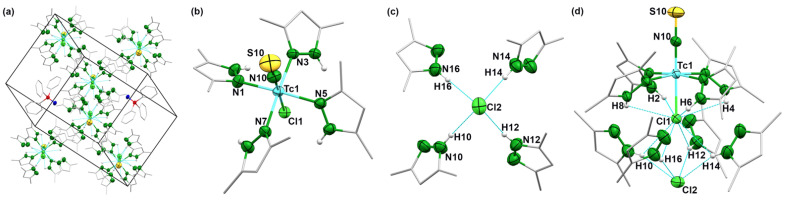
Solid state structure of **5**{Cl(pz^HMe2^)_4_}·OPPh_3_ showing (**a**) the content of the triclinic unit cell, (**b**) the [Tc(NS)Cl(pz^HMe2^)_4_]^+^ cation, (**c**) the·{Cl(pz^HMe2^)_4_}^−^ anion and (**d**) the H bond network within the [Tc(NS)Cl(pz^HMe2^)_4_]{Cl(pz^HMe2^)_4_} unit. Thermal ellipsoids show 50 per cent probability.

Figure 6 depicts the solid-state structure of **5**{Cl(pz^HMe2^)_4_}·OPPh_3_, in which [Tc(NS)Cl(pz^HMe2^)_4_]^+^ cations and {Cl(pz^HMe2^)_4_}^−^ anions form a hydrogen-bonded network with Cl2…H distances between 2.439 and 2.678 Å in the anion, while Cl1…H contacts between 2.687 and 2.904 Å are established. Details of this network are shown in Figure 6d and the individual atomic distances are listed in Table 4. The experimentally observed arrangement of the four dimethylpyrazole molecules around the chloride anion justifies treatment as a complex anionic {Cl(pz^HMe2^)_4_}^−^ unit. The co-crystallized triphenylphosphine oxide does not establish bonding interactions to the complex cation or the {Cl(pz^HMe2^)_4_}^−^ anions, as is clearly visible in Figure 6a. The bonding situation in the [Tc(NS)Cl(pz^HMe2^)_4_]^+^ cation is not exceptional, with the technetium atom in an only slightly distorted octahedral coordination environment. As in the other thionitrosyl complexes in this study, the Tc–N–S bond confirms the presence of a linear unit and justifies its treatment as an NS^+^ ligand.

The ready cleavage of the boron–nitrogen bonds of {HB(pz^R^)_3_}^−^ ligands during reactions with technetium complexes, even at room temperature, makes it interesting to study similar reactions with analogous rhenium compounds. The synthesis of a corresponding thionitrosylrhenium(II) complex, [Re(NS)Cl_3_(PPh_3_)_2_] [78], as a potential starting material has recently been reported and gives an opportunity to compare the reactivity of the two group 7 elements.

### 2.3. Thionitrosyl Complexes of Rhenium

**Scheme 4:**
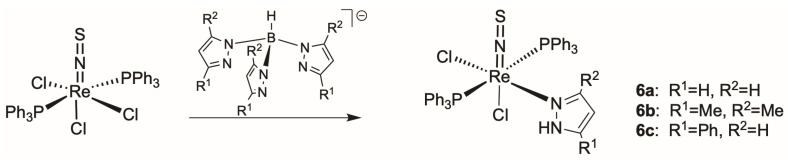
Reactions of [Re(NS)Cl_3_(PPh_3_)_2_] with hydrotris(pyrazolyl)borates and their products.

[Re(NS)Cl_3_(PPh_3_)_2_] is a dark red complex that is sufficiently soluble in CH_2_Cl_2_ to conduct reactions at room temperature. The treatment of a solution of the complex in this solvent with K{HB(pz)_3_} at room temperature results in a color change and an orange-brown solution is formed, from which a colorless solid (KCl) precipitates. After filtration and chromatography over a silica column, a green and a pale purple fraction can be isolated. The solids isolated from both fractions show identical IR spectra and solutions of the purple compound gradually change in color to green, which indicates a rapid conversion into this stable product of the reaction. It is formed as the sole product when the reaction mixture is heated under reflux. Finally, grayish green single crystals are obtained from such solutions. They have the composition [Re(NS)Cl_2_(PPh_3_)_2_(pz^H^)] (**6a**), and similar products were obtained when substituted hydrotris(pyrazole)borates were used (Scheme 4). 

No evidence was found for an ongoing ligand exchange or the formation of rhenium complexes with more than one pyrazole ligand. This is a clear difference to reactions of corresponding technetium compounds, where the corresponding 1:1 ligand exchange product was obtained for the nitrosyl complex, while the entire equatorial coordination sphere was occupied by pyrazole ligands in the thionitrosyl complex **5**. Lower reaction rates and consequently the isolation of products with “incomplete ligand exchange” is not unusual for couples of structurally equivalent *4d* and *5d* transition metal complexes and have also been observed for series of rhenium and technetium complexes, e.g., during the replacement of carbonyl ligands with isocyanides [94,95]. 

The formation of rhenium(I) complexes is not completely unexpected and is in line with the reduction of the [Tc(NS)Cl_3_(PPh_3_)_2_] starting material during the reaction with {HB(pz^Me2^)_3_}. The nature of the reducing agent, however, is not completely clear for both reactions. Most probably, it is related to the degradation of the tripodal ligand, but the oxidation of PPh_3_ also cannot be completely ruled out.

**Figure 7:**
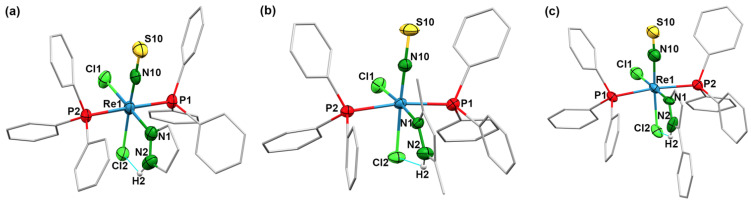
Molecular structures of (**a**) [Re(NS)Cl_2_(PPh_3_)_2_(pz^H^)] (**6a**), (**b**) [Re(NS)Cl_2_(PPh_3_)_2_(pz^HMe2^)] (**6b**) and (**c**) [Re(NS)Cl_2_(PPh_3_)_2_(pz^HPh^)] (**6c**). Thermal ellipsoids show 50 per cent probability.

Figure 7 depicts the structures of the [Re(NS)Cl_2_(PPh_3_)_2_(pz^HR^)] complexes. Selected bond lengths and angles are summarized in Table 5. The main structural features of the rhenium compounds are very similar and close to the bonding situation in the technetium complex **1**. The thionitrosyl units are essentially linear, as is expected for NS^+^ ligands. The rhenium atoms possess only slightly distorted octahedral coordination spheres with the chlorido ligands in trans positions to the thionitrosyl and the pyrazole ligands. Intramolecular hydrogen bonds are established between the heterocyles and the chlorido ligand trans to the NS^+^ group.

## 3. Materials and Methods

Unless otherwise stated, reagent-grade starting materials were purchased from commercial sources and either used as received or purified following standard procedures. Solvents were dried and distilled prior to use. [Tc(NO)Cl_2_(PPh_3_)_2_(CH_3_CN)] and [Re(NS)Cl_3_(PPh_3_)_2_] were prepared as described in the literature [26,78]. 

### 3.1. Radiation Precaution

All synthetic work with the long-lived isotope ^99^Tc was performed in a laboratory approved for the handling of radioactive material. The glass walls of the flasks provide appropriate protection from the primary beta emission of ^99^Tc. Secondary X-rays (bremsstrahlung) become important only when larger amounts of the compounds are handled as solids. All personnel working in this project were permanently monitored for potential contamination.

### 3.2. Syntheses

*[Tc(NO)Cl_2_(PPh_3_)_2_(pz^H^)]* (**1**). [Tc(NO)Cl_2_(PPh_3_)_2_(CH_3_CN)] (50 mg, 0.065 mmol) was suspended in 4 mL acetonitrile and solid K{HB(pz)_3_} (20 mg, 0.08 mmol) was added. The mixture was heated under reflux for 1 h. During this time, the sparingly soluble starting materials dissolved. The resulting red solution was filtered and the solvent was removed in vacuum. Red crystals were obtained by slow diffusion of MeOH into a CH_2_Cl_2_ solution of the product. Yield: 25 mg, 45%. IR (KBr, cm^−1^): 3315(w), 3055(w), 2918(w), 2851(w) 1708(vs, NO), 1481(w), 1435(m), 1259(w), 1130(w), 1091(m), 1053(w), 744(m), 694(s), 514(s). ^1^H NMR (CDCl_3_, ppm): 10.69 (br, 1H, NH), 7.43–7.38 (m, 12H, PPh_3_), 7.18–7.13 (m, 6H, PPh_3_), 7.09–7.05 (m, 12H, PPh_3_), 6.68 (d, 1H, pz), 6.54 (s, 1H, pz), 5.65 (s, 1H, pz). ^99^Tc NMR (CDCl_3_, ppm): 1217 (ν_1/2_ = 4000 Hz). 

*[Tc(NO)Cl(PPh_3_)(HB(pz)_3_}]* (**2**). [Tc(NO)Cl_2_(PPh_3_)_2_(pz^H^)] (25 mg, 0.03 mmol) was dissolved in 2 mL MeOH. K{HB(pz)_3_} and 5 drops of CH_2_Cl_2_ were added. The mixture was stirred for 3 h at room temperature under an argon atmosphere. An orange-brown solid deposited from this solution upon standing overnight in a refrigerator. This precipitate was dissolved in CH_2_Cl_2_ and overlayered with MeOH. Orange-brown crystals formed upon slow diffusion of the solvents. Yield: 14 mg, 75%. IR (KBr, cm^−1^): 3429(br), 3055(w), 2920(w), 2486(w), 2372(w), 1718(vs, NO), 1627(w), 1506(w), 1481(w), 1433(w), 1404(m), 1309(m), 1274(w), 1213(m), 1116(m), 1093(w), 1051(s), 989(w), 752(m), 696(m), 619(w), 526(m), 503(w). ^1^H NMR (CDCl_3_, ppm): 7.78 (d, 1H, pz), 7.68 (d, 1H, pz), 7.56 (d, 2H, pz), 7.39–7.26 (m, 15H, PPh_3_), 6.91 (d, 1H, pz), 6.27 (d, 1H, pz), 6.20 (t, 1H, pz), 5.89 (t, 1H, pz), 5.78 (t, 1H, pz). ^99^Tc NMR (CDCl_3_, ppm): 1019 (ν_1/2_ = 3400 Hz).

*[Tc(NS)Cl_3_(PPh_3_)_2_]* (**3**). (a) [TcNCl_2_(PPh_3_)_2_] (71 mg, 0.1 mmol) was suspended in 25 mL of dry CH_2_Cl_2_ under an atmosphere of dry argon. S_2_Cl_2_ (0.3 mL) was added and the mixture was stirred for 15 min at room temperature. The color of the mixture turned from orange-red to green. All volatiles were removed in vacuum and the resulting green residue was carefully washed with *n*-hexane and diethyl ether. The product was dissolved in CH_2_Cl_2_ under an atmosphere of dry argon and overlayered with *n*-pentane. Keeping this mixture in a refrigerator gave green crystals after diffusion of the solvents. Yield: 51 mg, 65%. (b) [Tc(NS)Cl_3_(PPh_3_)(OPPh_3_)] (39 mg, 0.05 mmol) was dissolved in 5 mL CH_2_Cl_2_ under an atmosphere of dry argon and PPh_3_ (130 mg, 0.5 mmol) was added. The mixture was heated under reflux for 30 min. The color of the solution changed from red to green. After concentration of the solution and addition of 100 mL cold diethyl ether, a green precipitate was formed. Filtration and crystallization from CH_2_Cl_2_/*n*-hexane gave green crystals. Yield: 33 mg, 85%. IR (KBr, cm^−1^): 2970(w), 2922(w), 2857(w), 1630(m) 1474(m), 1429(s), 1387(vs, NS), 1227(w), 1186(w), 1163(w), 1088(m), 1051(w), 988(w), 972(w), 914(w), 881(w), 810(w), 743(m), 692 (s), 605(w), 513(s), 455(w). EPR (RT, CH_2_Cl_2_) g_0_ = 2.011; a_0_^Tc^ = 164 × 10^−4^ cm^−1^. EPR (77 K, CH_2_Cl_2_): g_‖_ = 1.955, g_⊥_ = 2.0455; A_‖_^Tc^ = 270 × 10^−4^ cm^−1^, A_⊥_^Tc^ = 128 × 10^−4^ cm^−1^. 

*[Tc(NS)Cl_3_(PPh_3_)(OPPh_3_)]* (**4**). (a) [TcNCl_2_(PPh_3_)_2_] (50 mg, 0.07 mmol) was suspended in 15 mL CH_2_Cl_2_ and an excess of S_2_Cl_2_ (0.3 mL) was added. The mixture was heated under reflux in air. The sparingly soluble starting material gradually dissolved and the initially green solution changed its color to red. The progress of the reaction could be monitored by subsequent recording of EPR spectra. After the disappearance of the signals of compound **3**, which was normally observed after 30 min., all volatiles were removed in vacuum and the red residue was carefully washed with *n*-hexane and diethyl ether. Crystallization from CH_2_Cl_2_/*n*-hexane gave red crystals. Yield: 42 mg, 76%. (b) [Tc(NS)Cl_3_(PPh_3_)_2_] (39 mg, 0.05 mmol) was dissolved in CH_2_Cl_2_ and heated in air for approximately 30 min. The progress of the reaction could be monitored by EPR and red crystals of the product could be obtained as described above. Yield: 36 mg, 90%. IR (KBr, cm^−1^): 2053(w), 2968(m), 2922(m), 1630(m), 1578(w), 1476(m), 1433(s), 1393(m), 1315(w), 1244(s, NS), 1146 (vs, PO), 1126(vs), 1082(s), 933(w), 922(w), 880(w), 752(m), 721(s), 691(s), 536(vs). EPR (RT, CH_2_Cl_2_) g_0_ = 2.009; a_0_^Re^ = 166 × 10^−4^ cm^−1^. EPR (77 K, CH_2_Cl_2_): g_‖_ = 1.978, g_⊥_ = 1.999; A_‖_^Tc^ = 290 × 10^−4^ cm^−1^, A_⊥_^Tc^ = 134 × 10^−4^ cm^−1^. 

*[Tc(NS)Cl(pz^H^)_4_]{Cl(pz^H^)_4_} (***5***{Cl(pz^H^)_4_}).* [Tc(NS)Cl_3_(PPh_3_)_2_] (50 mg, 0.06 mmol) was dissolved in 10 mL CH_2_Cl_2_ and a solution of K{HB(pz^HMe2^)_3_} (143 mg, 0.12 mmol) in 3 mL MeOH was added. The color of the mixture immediately changed from red to brown. After stirring for 6 h at room temperature, all volatiles were removed in vacuum and the residue was washed with *n*-hexane and extracted with 5 mL CH_2_Cl_2_. *N*-hexane (5 mL) was added and pale blue single crystals were obtained after storing the mixture in a refrigerator. Yield: 24 mg, 38%. IR (KBr, cm^−1^): 3171(m), 3127(m), 3063(m), 3022(m), 3953(s), 2922(s), 2772(s), 2713(s), 2658(s), 2583(m), 2529(s), 2372(m), 2284(m), 1647(w), 1599(vs), 1558(s), 1518(w), 1468(w), 1410(vs), 1371(w), 1290 (vs, NS), 1161(vs), 1119(s), 1024(s), 986(m), 849(vs), 806(m), 758(w), 723(s), 689(s), 586(s), 536(vs), 482(w). ^1^H NMR (CDCl_3_, ppm): 10.53 (s, 8H, NH), 6.10 (s, 4H, CH complex), 5.95 (s, 4H, CH anion), 2.40 (s, 12H, CH_3_ complex), 1.91 (s, 12H, CH_3_ anion). ^99^Tc NMR (CDCl_3_, ppm): +566 (ν_1/2_ = 1860 Hz).

*[Re(NS)Cl_2_(PPh_3_)_2_(pz^H^)]* (**6a**). [Re(NS)Cl_3_(PPh_3_)_2_] (100 mg, 0.12 mmol) was dissolved in 30 mL CH_2_Cl_2_ and a suspension of K{HB(pz)_3_} in 20 mL CH_2_Cl_2_ was added. The mixture was heated under reflux for 1 h. The color of the solution changed to greenish yellow and a colorless solid precipitated. After filtration, the volume of the reaction mixture was reduced to 10 mL and the same amount of *n*-hexane was added. A pale green solid formed upon slow concentration of this solution. The crude product was purified by chromatography via a silica column with a CH_2_Cl_2_/*n*-hexane mixture (9:1, *v*/*v*) as mobile phase. The major product was first eluted as a green fraction, followed by a pale purple fraction. Concentration of the green fraction gave a microcrystalline product. Yield: 85 mg, 61%. Elemental analysis: Calcd. for C_39_H_34_Cl_2_N_3_P_2_ReS: C, 52.3; H, 3.8; N, 4.7; S, 3.6%. Found: C 53.0; H, 4.9; N, 4.9; S, 3.1%. IR (ATR, cm^−1^): 3273(w), 1481(m), 1432(s), 1339(m), 1259(s, NS), 1171(vs), 1154(m), 1128(s), 1090(s), 1046(s), 997(w), 913(w), 767(w), 741(s), 691(vs), 617(w), 598(m), 589(w). ^1^H NMR (CD_2_Cl_2_, ppm): 10.73 (s, 1H, NH); 7.61–7.22 (m, 30H, PPh_3_); 7.03 (t, 1H, pz); 6.98 (t, 1H, pz); 5.44 (dd, 1H, pz). ^31^P NMR (CD_2_Cl_2_, ppm): –1.6 (s). ESI+ MS: *m*/*z* = 918.095 [M+Na]^+^ (calcd. 918.077), 934.067 [M+K]^+^ (calcd. 934.052).

*[Re(NS)Cl_2_(PPh_3_)_2_(pz^HMe2^)]* (**6b**). The compound was prepared and purified as described for complex **6a** with K{HB(pz^Me2^)_3_}. Grey-green needles. Yield: 35 mg, 33%. Elemental analysis: Calcd. for C_41_H_38_Cl_2_N_3_P_2_ReS: C, 53.3; H, 4.2; N, 4.6; S, 4.6%. Found: C 53.2; H, 3.9; N, 4.4; S, 4.3%. IR (ATR, cm^−1^): 3280(w), 1570(w), 1481(w), 1433(m), 1273(m, NS), 1174(s), 1093(m), 1028(w), 1020(w), 918(w), 815(m), 748(m), 692(vs). ^1^H NMR (CD_2_Cl_2_, ppm): 10.45 (s, 1H, NH); 7.63–7.60 (m, 12H, PPh_3_); 7.29–7.25 (m, 18H, PPh_3_); 1.87 (s, 3H, CH_3_); 1.84 (s, 3H, CH_3_). ^31^P NMR (CD_2_Cl_2_, ppm): −3.4 (s). ESI+ MS: *m*/*z* = 923.119 [M]+ (calcd. 923.120), 946.109 [M+Na]^+^ (ber.: 946.108), 962.089 [M+K]^+^ (calcd. 962.082), 1869.229 [2M+Na]^+^ (calcd. 1869.223), 1885.204 [2M+K]^+^ (calcd. 1885.197).

*[Re(NS)Cl_2_(PPh_3_)_2_(pz^HPh^)]* (**6c**). The compound was prepared as described for compound **6a** with K{HB(pz^Ph^)_3_}. The workup was performed without column chromatography via crystallization from CHCl_3_. Pale green crystals. Yield: 82 mg, 73%. Elemental analysis: Calcd. for C_45_H_38_Cl_2_N_3_P_2_ReS: C, 55.6; H, 3.9; N, 4.3; S, 4.3%. Found: C 55.3; H, 3.8; N, 4.2; S, 3.9%. IR (ATR, cm^−1^): 3276(w), 3061(w), 1586(w), 1574(w), 1558(w), 1481(m), 1435(s), 1349(w), 1267(w, NS), 1182(m), 1170(s) 1126(m), 1091(s), 1027(w), 998(w), 959 (w), 919(m), 849(w), 801(m), 469(m), 741(s), 690(vs), 658(s), 617(m). ^1^H NMR (CD_2_Cl_2_): 11.01 (s, 1H, NH), 7.63–7.71 (m, 12H, Ph(PPh_3_)), 7.28–7.37 (m, 3H, Ph(pz)), 7.22–7.30 (m, 18H, Ph(PPh3)), 6.94–7.01 (m, 2+1H, Ph(pz), CH), 6.00 (s, 1H, CH). ^31^P NMR (CD_2_Cl_2_, ppm): –2.3 (s). ESI+ MS: *m*/*z* = 971.145 [M]^+^ (calcd. 971.119), 994.136 [M+Na]^+^ (calcd. 994.108), 1010.112 [M+K]^+^ (calcd. 1010.082).

### 3.3. Spectroscopic and Analytical Methods

IR spectra were measured as KBr pellets on a Shimadzu IR Affinity-1 spectrometer (Shimadzu, Kyoto, Japan) (technetium compounds) or with a Thermo Scientific Nicolet iS10 ATR spectrometer (ThermoFisher Scientific, Madison, WI, USA) (rhenium complexes). The NMR spectra were recorded on JEOL 400 MHz spectrometers (JEOL, Kyoto, Japan). An aqueous solution of KTcO_4_ was used as reference for the ^99^Tc spectra. ESI mass spectra were measured with an Agilent 6210 ESI-TOF (Agilent Technology, Santa Clara, CA, USA) mass spectrometer. All MS results are given in the form of *m/z* assignment. Elemental analysis of carbon, hydrogen, nitrogen, and sulfur were determined using a Heraeus vario EL elemental analyzer (Elementar, Langensebold, Germany). Combustion analyses could not be conducted for the radioactive technetium compounds for radiation protection reasons.

### 3.4. X-ray Crystallography

The intensities for the X-ray determinations were collected on a STOE IPDS-2T (STOE, Darmstadt, Germany) with Mo/Kα radiation. Numerical absorption corrections were carried out by X-RED32 [96]. Structure solution and refinement were performed with the SHELX programs [97,98] included in the WinGX [99] program package or OLEX2 [100]. Hydrogen atoms were calculated for the idealized positions and treated with the ‘riding model’ option of SHELXL. Since some of the compounds crystallized together with disordered solvent molecules (partially close to special positions), refinements of such structures were undertaken with the removal of disordered solvent molecules using the solvent mask option of OLEX2. Details are given in the Supplementary Materials. The representation of molecular structures was prepared using the program MERCURY [101].

## 4. Conclusions

Most of the conducted reactions of common nitrosyl and thionitrosyl complexes of technetium and rhenium with hydrotris(pyrazolyl)borate ligands result in B–N bond cleavage and the formation of pyrazole complexes. The number of pyrazole ligands in the products is variable and ranges from one (in most of the studied cases) to four in the thionitrosyl cation [Tc(NS)Cl(pz^HMe2^)_4_]^+^. In only one exceptional case, the reaction of [Tc(NO)Cl_2_(PPh_3_)_2_(pz^H^)] with K{HB(pz)_3_} at room temperature, was the coordination of an intact hydrotris(pyrazolyl)borato ligand observed. Generally, it can be concluded that any thermal stress to such reaction mixtures results in degradation of the respective ligand systems. The decomposition is most probably metal-driven or at least metal-supported, since the uncoordinated ligands, e.g., K{HB(pz)_3_} or K{HB(pz^Me2^)_3_}, are synthesized in melts at temperatures >200 °C [102,103].

## Figures and Tables

**Table 1:** Selected bond lengths (Å) and angles in [Tc(NO)Cl_2_(PPh_3_)2(pz^H^)] (**1**) and [Tc(NO)Cl(PPh_3_){HB(pz)_3_}] (**2**).

	Tc1–N10	N10–O10	Tc1–Cl1	Tc1–Cl2	Tc1–P1	Tc1–P2	Tc1–N1	Tc1–N3	Tc1–N5	Tc1–N10–O10
**1**	1.816(4)	0.946(4)	2.447(1)	2.443(2)	2.474(2)	2.473(1)	2.4149(4)	-	-	178.4(5)
**2**	1.764(7)	1.13(1)	2.425(2)	-	2.4417(5)	-	2.152(2)	2.172(1)	2.135(2)	174.3(5)

**Table 2:** EPR parameters of **3** and **4** together with the values for some other nitrosyl and thionitrosyl complexes of Tc(II). Coupling constants are given in 10^−4^ cm^−1^.

	g_0_	a_0_^Tc^	g_‖_	g_⊥_	A_‖_^Tc^	A_⊥_^Tc^	A_‖_^P^	Reference
[Tc(NS)Cl_3_(PPh_3_)_2_] (**3**)	2.011	164	1.955	2.0455	270	128	-	This work
[Tc(NS)Cl_3_(PPh_3_)(OPPh_3_)] (**4**)	2.009	166	1.978	1.999	290	134	-	This work
[Tc(NS)Cl_3_(PMe_2_Ph)_2_]	2.045	133	2.027	2.038	219	101	19 ^(a)^	[70]
[Tc(NS)Cl_3_(PMe_2_Ph)(OPMe_2_Ph)]	2.032	149	2.027	2.039	237	106	24 ^(b)^	[67]
[Tc(NO)Cl_3_(PMe_2_Ph)_2_]	2.045	125	2.034	2.053	215	88	19 ^(a)^	[93]

^(a)^ Triplet, ^(b)^ Doublet.

**Table 3:** Selected bond lengths (Å) and angles in [Tc(NS)Cl_3_(PPh_3_)_2_] (**3**) and [Tc(NS)Cl_3_(PPh_3_)(OPPh_3_)] (**4**).

	Tc1–N10	N10–S10	Tc1–Cl1	Tc1–Cl2	Tc1–Cl3	Tc1–P1/P2	Tc1–O1	P1–O1	Tc1–O1–P1	Tc1–N10–S10
**3**	1.78(1)	1.51(1)	2.443(3)	2.357(2)	-	2.570(2)	-	-	-	180
**4**	1.68(2)	1.55(2)	2.346(6)	2.368(6)	2.356(6)	2.552(7)	2.09(1)	1.50(1)	154.4(9)	174(2)

**Table 4:** Selected bond lengths (Å) and angles in the solid state structure of [Tc(NS)Cl(pz^HMe2^)_4_]{Cl(pz^HMe2^)_4_}·OPPh_3_ (**5**{Cl(pz^HMe2^)_4_}·OPPh_3_).

Tc1–N10	N10–S10		Tc1–Cl1		Tc1–N1		Tc1–N3		Tc1–N5		Tc1–N7		Tc1–N10–S10		N10–Tc1–Cl1
1.733(2)	1.572(2)		2.4252(6)		2.134(2)		2.135(2)		2.147(2)		2.137(2)		179.4(2)		179.66(8)
Hydrogen Bonds															
**H10–Cl1**		**H12–Cl1**		**H14–Cl1**		**H16–Cl1**		**H2–Cl1**		**H4–Cl1**		**H6–Cl1**		**H8–Cl1**	
2.851		2.687		2.851		2.904		2.867		2.864		2.868		2.825	
**H10–Cl2**		**H12–Cl2**		**H14–Cl2**		**H16–Cl2**									
2.507		2.678		2.478		2.439									

**Table 5:** Selected bond lengths (Å) and angles in [Re(NS)Cl_2_(PPh_3_)_2_(pz^H^)] (**6a**), [Re(NS)Cl_2_(PPh_3_)_2_(pz^HMe2^)] (**6b**) and [Re(NS)Cl_2_(PPh_3_)_2_(pz^HPh^)] (**6c**).

	Re–N10	N10–S10	Re1–Cl1	Re1–Cl2	Re1–N1	Cl2…H2	Re1–N10–S10	N10–Re1–Cl2	N2–H2…Cl2
**6a**	1.738(8)	1.558(7)	2.438(2)	2.488(4)	2.142(5)	2.536	178.3(3)	174.8(2)	118.9
**6b**	1.737(5)	1.578(5)	2.445(2)	2.468(1)	2.158(4)	2.396	173.0(3)	176.6(2)	125.5
**6c**	1.98(2)	1.36(2)	2.441(8)	2.449(4)	2.18(2)	2.501	175(2)	171(1)	124.4

## Data Availability

The original contributions presented in the study are included in the article/Supplementary Materials, further inquiries can be directed to the corresponding author.

## Supplementary Materials

The following supporting information can be downloaded at: https://www.mdpi.com/article/10.3390/molecules29163865/s1: Table S1.1: Crystallographic data and data collection parameters. Figure S1.1. Ellipsoid representation of the structure of [Tc(NO)Cl_2_(PPh_3_)_2_(pz^H^)] (**1**)·CH_2_Cl_2_. The thermal ellipsoids are set at a 50% probability level. Hydrogen atoms are omitted for clarity. Table S1.2. Selected bond lengths (Å) and angles (°) in [Tc(NO)Cl_2_(PPh_3_)_2_(pz^H^)] (**1**). Figure S1.2. Ellipsoid representation of the molecular structure of [Tc(NO)Cl(PPh_3_){HB(pz)_3_}] (**2**) including the positional disorder between the Tc–Cl and Tc–N–O bonds. The thermal ellipsoids are set at a 50% probability level. Hydrogen atoms are omitted for clarity. Table S1.3. Selected bond lengths (Å) and angles (°) in [Tc(NO)Cl(PPh_3_){HB(pz)_3_}] (**2**). Figure S1.3. Ellipsoid representation of [Tc(NS)Cl_3_(PPh_3_)_2_] (**3**). The thermal ellipsoids are set at a 50% probability level. Hydrogen atoms are omitted for clarity. Table S1.4. Selected bond lengths (Å) and angles (°) in [Tc(NS)Cl_3_(PPh_3_)_2_] (**3**). Figure S1.4. Ellipsoid representation of [Tc(NS)Cl_3_(PPh_3_)(OPPh_3_)] (**4**) including the positional disorder between the Tc–N–S and Tc–Cl bonds. The thermal ellipsoids are set at a 50% probability level. Hydrogen atoms are omitted for clarity. Table S1.5. Selected bond lengths (Å) and angles (°) in [Tc(NS)Cl_3_(PPh_3_)(OPPh_3_)] (**4**). Figure S1.5. Ellipsoid representation of [Tc(NS)Cl(pz^HMe2^)_4_]{Cl(pz^HMe2^)_4_} (**5**{Cl(pz^HMe2^)_4_})⋅(OPPh_3_). The thermal ellipsoids are set at a 50% probability level. Table S1.6. Selected bond lengths (Å) and angles (°) in [Tc(NS)Cl(pz^HMe2^)_4_]{Cl(pz^HMe2^)_4_} (**5**{Cl(pz^HMe2^)_4_})·(OPPh_3_). Figure S1.6. Ellipsoid representation of [Re(NS)Cl_2_(PPh_3_)_2_(pz^H^)] (**6a**)·0.5(CH_2_Cl_2_) including positional disorder for the NO and Cl_3_ ligands of the central molecular axis. The thermal ellipsoids are set at a 50% probability level. Table S1.7. Selected bond lengths (Å) and angles (°) in [Re(NS)Cl_2_(PPh_3_)_2_(pz^H^)] (**6a**)·0.5(CH_2_Cl_2_). Figure S1.7. Ellipsoid representation of [Re(NS)Cl_2_(PPh_3_)_2_(pz^HMe2^)] (**6b**). The thermal ellipsoids are set at a 50% probability level. Table S1.8. Selected bond lengths (Å) and angles (°) [Re(NS)Cl_2_(PPh_3_)_2_(pz^HMe2^)] (**6b**). Figure S1.8. Ellipsoid representation of [Re(NS)Cl_2_(PPh_3_)_2_(pz^HPh^)] (**6c**) including positional disorder between the NS and the Cl_1_ atom ligands. The thermal ellipsoids are set at a 50% probability level. Table S1.9. Selected bond lengths (Å) and angles (°) in [Re(NS)Cl_2_(PPh_3_)_2_(pz^HPh^)] (**6c**). Figure S2.1: IR (KBr) spectrum of [Tc(NO)Cl_2_(PPh_3_)_2_(pz^H^)] (**1**). Figure S2.2: ^1^H NMR spectrum of [Tc(NO)Cl_2_(PPh_3_)_2_(pz^H^)] (**1**) in CDCl_3_. Figure S2.3: ^31^P NMR spectrum of [Tc(NO)Cl_2_(PPh_3_)_2_(pz^H^)] (**1**) in CDCl_3_. Figure S2.3: ^99^Tc NMR spectrum of [Tc(NO)Cl_2_(PPh_3_)_2_(pz^H^)] (**1**) in CDCl_3_. Figure S2.4: IR (KBr) spectrum of [Tc(NO)Cl(PPh_3_){HB(pz)_3_}] (**2**). Figure S2.5: ^1^H NMR spectrum of [Tc(NO)Cl(PPh_3_){HB(pz)_3_}] (**2**) in CDCl_3_. Figure S2.6: ^31^P NMR spectrum of [Tc(NO)Cl(PPh_3_){HB(pz)_3_}] (**2**) in CDCl_3_. Figure S2.7: ^99^Tc NMR spectrum of [Tc(NO)Cl(PPh_3_){HB(pz)_3_}] (**2**) in CDCl_3_. Figure S2.8: IR (KBr) spectrum of [Tc(NS)Cl_3_(PPh_3_)_2_] (**3**). Figure S2.9: Solution EPR spectrum of [Tc(NS)Cl_3_(PPh_3_)_2_] (**3**) in CH_2_Cl_2_ at room temperature. Figure S2.10: Solution EPR spectra of [Tc(NS)Cl_3_(PPh_3_)_2_] (**3**) in CH_2_Cl_2_ at T = 77 K. Figure S2.11: IR (KBr) spectrum of [Tc(NS)Cl_3_(PPh_3_)(OPPh_3_)] (**4**). Figure S2.12: Solution EPR spectrum of [Tc(NS)Cl_3_(PPh_3_)(OPPh_3_)] (**4**) in CH_2_Cl_2_ at room temperature. Figure S2.13: Solution EPR spectra of [Tc(NS)Cl_3_(PPh_3_)(OPPh_3_)] (**4**) in CH_2_Cl_2_ at T = 77 K. Figure S2.14: IR (KBr) spectrum of [Tc(NS)Cl(pz^HMe2^)_4_]{Cl(pz^HMe2^)_4_} (**5**{Cl(pz^HMe2^)_4_})·(OPPh_3_). Figure S2.15: ^1^H NMR spectrum of [Tc(NS)Cl(pz^HMe2^)_4_]{Cl(pz^HMe2^)_4_} (**5**{Cl(pz^HMe2^)_4_})·(OPPh_3_) in CDCl_3_. Figure S2.16: ^99^Tc NMR spectrum of [Tc(NS)Cl(pz^HMe2^)_4_]{Cl(pz^HMe2^)_4_} (**5**{Cl(pz^HMe2^)_4_})·(OPPh_3_) in CDCl_3_. Figure S2.17: IR (ATR) spectrum of [Re(NS)Cl_2_(PPh_3_)_2_(pz^H^)] (**6a**). Figure S2.18: ^1^H NMR spectrum of [Re(NS)Cl_2_(PPh_3_)_2_(pz^H^)] (**6a**) in CD_2_Cl_2_. Figure S2.19: ^31^P NMR spectrum of [Re(NS)Cl_2_(PPh_3_)_2_(pz^H^)] (**6a**) in CD_2_Cl_2_. Figure S2.20: ESI+ mass spectrum of [Re(NS)Cl_2_(PPh_3_)_2_(pz^H^)] (**6a**). Figure S2.21: IR (ATR) spectrum of [Re(NS)Cl_2_(PPh_3_)_2_(pz^HMe2^)] (**6b**). Figure S2.22: ^1^H NMR spectrum of [Re(NS)Cl_2_(PPh_3_)_2_(pz^HMe2^)] (**6b**) in CD_2_Cl_2_. Figure S2.23: ^31^P NMR spectrum of [Re(NS)Cl_2_(PPh_3_)_2_(pz^HMe2^)] (**6b**) in CD_2_Cl_2_. Figure S2.24: ESI+ mass spectrum of [Re(NS)Cl_2_(PPh_3_)_2_(pz^HMe2^)] (**6b**). Figure S2.25: IR (ATR) spectrum of [Re(NS)Cl_2_(PPh_3_)_2_(pz^HPh^)] (**6c**). Figure S2.26: ^1^H NMR spectrum of [Re(NS)Cl_2_(PPh_3_)_2_(pz^HPh^)] (**6c**) in CD_2_Cl_2_. Figure S2.27: ^31^P NMR spectrum of [Re(NS)Cl_2_(PPh_3_)_2_(pz^HPh^)] (**6c**) in CD_2_Cl_2_. Figure S2.28: ESI+ mass spectrum of [Re(NS)Cl_2_(PPh_3_)_2_(pz^HPh^)] (**6c**).
