# Supplementary material for: Nitrosyl and Thionitrosyl Complexes of Technetium and Rhenium and Their Reactions with Hydrotris(pyrazolyl)borates
Source: Molecules. 2024 Aug 15;29(16):3865. doi: 10.3390/molecules29163865 (PMC11357682; doi:10.3390/molecules29163865)
Supplement: Supplementary file 1 [file molecules-29-03865-s001.zip › molecules-3159243-supplementary.pdf]

Supplementary Materials to the paper entitled:

Nitrosyl and Thionitrosyl Complexes of Technetium and Rhenium and Their Reactions with Hydrotris(pyrazolylborates)

Till Erik Sawallisch, Abdullah Abdulkader, Domenik Nowak, Adelheid Hagenbach, and Ulrich Abram*

Institute of Chemistry and Biochemistry, Freie Universität Berlin, Fabeckstr. 34/36, 14195 Berlin, Germany.

Table of content

1. Crystallographic data 4

Table S1.1: Crystallographic data and data collection parameters 4

Figure S1.1. Ellipsoid representation of the structure of $[Tc(NO)Cl_2(PPh_3)_2(pz^H)]$ (**1**) · CH_2Cl_2. The thermal ellipsoids are set at a 50% probability level. Hydrogen atoms are omitted for clarity. 8

Table S1.2. Selected bond lengths (Å) and angles (°) in $[Tc(NO)Cl_2(PPh_3)_2(pz^H)]$ (**1**). 8

Figure S1.2. Ellipsoid representation of the molecular structure of $[Tc(NO)Cl(PPh_3)\{HB(pz)_3\}]$ (**2**) including the positional disorder between the Tc-Cl and Tc–N–O bonds. The thermal ellipsoids are set at a 50% probability level. Hydrogen atoms are omitted for clarity. 9

Table S1.3. Selected bond lengths (Å) and angles (°) in $[Tc(NO)Cl(PPh_3)\{HB(pz)_3\}]$ (**2**). .. 9

Figure S1.3. Ellipsoid representation of $[Tc(NS)Cl_3(PPh_3)_2]$ (**3**). The thermal ellipsoids are set at a 50% probability level. Hydrogen atoms are omitted for clarity. 10

Table S1.4. Selected bond lengths (Å) and angles (°) in $[Tc(NS)Cl_3(PPh_3)_2]$ (**3**). 10

Figure S1.4. Ellipsoid representation of $[Tc(NS)Cl_3(PPh_3)(OPPh_3)]$ (**4**) including the positional disorder between the Re–N–S and Re–Cl bonds. The thermal ellipsoids are set at a 50% probability level. Hydrogen atoms are omitted for clarity. 11

Table S1.5. Selected bond lengths (Å) and angles (°) in $[Tc(NS)Cl_3(PPh_3)(OPPh_3)]$ (**4**). .. 11

Table S1.6. Selected bond lengths (Å) and angles (°) in $[Tc(NS)Cl(pz^{HMe2})_4]\{Cl(pz^{HMe2})_4\}$ (**5**$\{Cl(pz^{HMe2})_4\}$) · $(OPPh_3)$. 12

Figure S1.6. Ellipsoid representation of $[Re(NS)Cl_2(PPh_3)_2(pz^H)]$ (**6a**) · $0.5(CH_2Cl_2)$ including the positional disorder for the NO and Cl3 ligands of the central molecular axis. The thermal ellipsoids are set at a 50% probability level 13

Table S1.7. Selected bond lengths (Å) and angles (°) in $[Re(NS)Cl_2(PPh_3)_2(pz^H)]$ (**6a**) · $0.5(CH_2Cl_2)$ 13

Figure S1.7. Ellipsoid representation of $[Re(NS)Cl_2(PPh_3)_2(pz^{HMe2})]$ (**6b**). The thermal ellipsoids are set at a 50% probability level. 14

Table S1.8. Selected bond lengths (Å) and angles (°) $[Re(NS)Cl_2(PPh_3)_2(pz^{HMe2})]$ (**6b**) 14

Figure S1.8. Ellipsoid representation of $[Re(NS)Cl_2(PPh_3)_2(pz^{HPh})]$ (**6c**) including the positional disorder between the NS and the Cl1 ligands. The thermal ellipsoids are set at a 50% probability level 15

Table S1.9. Selected bond lengths (Å) and angles (°) in $[Re(NS)Cl_2(PPh_3)_2(pz^{HPh})]$ (**6c**) .. 15

2. Spectroscopic data 16

Figure S2.1: IR (KBr) spectrum of $[Tc(NO)Cl_2(PPh_3)_2(pz^H)]$ (**1**) 16

Figure S2.2: ^1H NMR spectrum of $[Tc(NO)Cl_2(PPh_3)_2(pz^H)]$ (**1**) in $CDCl_3$. 16

Figure S2.3: ^{31}P NMR spectrum of [Tc(NO)Cl_2(PPh_3)$_2$(pz^H)] (**1**) in $CDCl_3$. 16

Figure S2.3: ^{99}Tc NMR spectrum of [Tc(NO)Cl_2(PPh_3)$_2$(pz^H)] (**1**) in $CDCl_3$. 17

Figure S2.4: IR (KBr) spectrum of [Tc(NO)Cl(PPh_3){HB(pz)$_3$)}] (**2**). 17

Figure S2.5: ^{1}H NMR spectrum of [Tc(NO)Cl(PPh_3){HB(pz)$_3$}] (**2**) in $CDCl_3$. 17

Figure S2.6: ^{31}P NMR spectrum of [Tc(NO)Cl(PPh_3){HB(pz)$_3$}] (**2**) in $CDCl_3$. 18

Figure S2.7: ^{99}Tc NMR spectrum of [Tc(NO)Cl(PPh_3){HB(pz)$_3$}] (**2**) in $CDCl_3$. 18

Figure S2.8: IR (KBr) spectrum of [Tc(NS)Cl_3(PPh_3)$_2$] (**3**). 18

Figure S2.9: Solution EPR spectrum of [Tc(NS)Cl_3(PPh_3)$_2$] (**3**) in CH_2Cl_2 at room-temperature. 19

Figure S2.10: Solution EPR spectra of [Tc(NS)Cl_3(PPh_3)$_2$] (**3**) in CH_2Cl_2 at T = 77 K. 19

Figure S2.11: IR (KBr) spectrum of [Tc(NS)Cl_3(PPh_3)($OPPh_3$)] (**4**). 19

Figure S2.12: Solution EPR spectrum of [Tc(NS)Cl_3(PPh_3)($OPPh_3$)] (**4**) in CH_2Cl_2 at room-temperature. 20

Figure S2.13: Solution EPR spectra of [Tc(NS)Cl_3(PPh_3)($OPPh_3$)] (**4**) in CH_2Cl_2 at T = 77 K. 20

Figure S2.14: IR (KBr) spectrum of [Tc(NS)Cl(pz^{HMe2})$_4$]{Cl(pz^{HMe2})$_4$} (**5**{Cl(pz^{HMe2})$_4$}) · ($OPPh_3$). 20

Figure S2.15: ^{1}H NMR spectrum of [Tc(NS)Cl(pz^{HMe2})$_4$]{Cl(pz^{HMe2})$_4$} (**5**{Cl(pz^{HMe2})$_4$}) · ($OPPh_3$) in $CDCl_3$. 21

Figure S2.16: ^{99}Tc NMR spectrum of [Tc(NS)Cl(pz^{HMe2})$_4$]{Cl(pz^{HMe2})$_4$} (**5**{Cl(pz^{HMe2})$_4$}) · ($OPPh_3$) in $CDCl_3$. 21

Figure S2.17: IR (ATR) spectrum of [Re(NS)Cl_2(PPh_3)$_2$(pz^H)] (**6a**). 21

Figure S2.18: ^{1}H NMR spectrum of [Re(NS)Cl_2(PPh_3)$_2$(pz^H)] (**6a**) in CD_2Cl_2. 22

Figure S2.19: ^{31}P NMR spectrum of [Re(NS)Cl_2(PPh_3)$_2$(pz^H)] (**6a**) in CD_2Cl_2. 22

Figure S2.20: ESI+ mass spectrum of [Re(NS)Cl_2(PPh_3)$_2$(pz^H)] (**6a**). 22

Figure S2.21: IR (ATR) spectrum of [Re(NS)Cl_2(PPh_3)$_2$(pz^{HMe2})] (**6b**). 23

Figure S2.22: ^{1}H NMR spectrum of [Re(NS)Cl_2(PPh_3)$_2$(pz^{HMe2})] (**6b**) in CD_2Cl_2. 23

Figure S2.23: ^{31}P NMR spectrum of [Re(NS)Cl_2(PPh_3)$_2$(pz^{HMe2})] (**6b**) in CD_2Cl_2. 23

Figure S2.24: ESI+ mass spectrum of [Re(NS)Cl_2(PPh_3)$_2$(pz^{HMe2})] (**6b**). 24

Figure S2.25: IR (ATR) spectrum of [Re(NS)Cl_2(PPh_3)$_2$(pz^{HPh})] (**6c**). 24

Figure S2.26: ^{1}H NMR spectrum of [Re(NS)Cl_2(PPh_3)$_2$(pz^{HPh})] (**6c**) in CD_2Cl_2. 24

Figure S2.27: ^{31}P NMR spectrum of [Re(NS)Cl_2(PPh_3)$_2$(pz^{HPh})] (**6c**) in CD_2Cl_2. 25

Figure S2.28: ESI+ mass spectrum of [Re(NS)Cl_2(PPh_3)$_2$(pz^{HPh})] (**6c**). 25

1. Crystallographic data

Table S1.1: Crystallographic data and data collection parameters.

	$[Tc(NO)Cl_2(PPh_3)_2(pz^H)]$ (**1**) · CH_2Cl_2	$[Tc(NO)Cl(PPh_3)\{HB(pz)_3\}]$ (**2**)
Empirical formula	$C_{40}H_{36}Cl_4N_3OP_2Tc$	$C_{27}H_{325}BClN_7OPTc$
Formula weight	876.46	638.77
Temperature/K	293(2)	230(2)
Crystal system	Monoclinic	Monoclinic
Space group	$P2_1/n$	C2/c
a/Å	13.998(1)	31.212(1)
b/Å	13.861(1)	9.7074(2)
c/Å	19.723(2)	18.5091(8)
α/°	90	90
β/°	94.688(7)	92.903(3)
γ/°	90	90
Volume/Å^3	3813.9(5)	5600.8(3)
Z	4	8
ρ_{calc} / gcm^{-3}	1.526	1.515
μ / mm^{-1}	0.779	0.701
F(000)	1784.0	2592.0
Crystal size / mm^3	0.20 × 0.15 × 0.05	0.50 × 0.31 × 0.16
Radiation	MoKα (λ = 0.71073)	MoKα (λ = 0.71073)
2Θ range for data collection/°	6.538 to 52	9.782 to 53.998
Index ranges	-17 ≤ h ≤ 17, -17 ≤ k ≤ 17, -20 ≤ l ≤ 24	-39 ≤ h ≤ 39, -11 ≤ k ≤ 12, -22 ≤ l ≤ 23
Reflections collected	32867	29163
Independent reflections	7478 [R_{int} = 0.0827, R_{sigma} = 0.1011]	6073 [R_{int} = 0.0505, R_{sigma} = 0.0350]
Data/restraints/parameters	7478/18/449	6073/0/383
Goodness-of-fit on F^2	0.808	0.952
Final R indexes [I>=2σ (I)]	R_1 = 0.0500, wR_2 = 0.1145	R_1 = 0.0250, wR_2 = 0.0555
Final R indexes [all data]	R_1 = 0.1211, wR_2 = 0.1318	R_1 = 0.0374, wR_2 = 0.0583
Largest diff. peak/hole / e Å^{-3}	0.70/-1.10	0.30/-0.39
Diffractometer	STOE IPDS T2	STOE IPDS T2
Remarks	-	-
CCDC access code	2370201	2370202

Table S1.1: Crystallographic data and data collection parameters (continued)

	$[Tc(NS)Cl_3(PPh_3)_2]$ (**3**)	$[Tc(NS)Cl_3(PPh_3)(OPPh_3)]$ (**4**)
Empirical formula	$C_{36}H_{30}Cl_3NP_2STc$	$C_{36}H_{30}Cl_3NOP_2STc$
Formula weight	774.96	790.96
Temperature/K	200(2)	200(2)
Crystal system	Monoclinic	Triclinic
Space group	C2/c	P-1
a/Å	24.862(7)	10.228(2)
b/Å	9.412(2)	17.973(4)
c/Å	16.211(5)	20.178(5)
α/°	90	90.11(1)
β/°	117.07(3)	91.01(2)
γ/°	90	94.45(2)
Volume/Å^3	3378(2)	3719(1)
Z	4	4
ρ_{calc} / gcm^{-3}	1.524	1.413
μ / mm^{-1}	0.848	0.773
F(000)	1572.0	1604.0
Crystal size / mm^3	0.26 × 0.26 × 0.18	0.30 × 0.17 × 0.03
Radiation	MoKα (λ = 0.71073)	MoKα (λ = 0.71073)
2Θ range for data collection/°	9.86 to 49.996	6.61 to 52
Index ranges	-29 ≤ h ≤ 29, -11 ≤ k ≤ 9, -19 ≤ l ≤ 19	-12 ≤ h ≤ 11, -22 ≤ k ≤ 20, -24 ≤ l ≤ 24
Reflections collected	11403	30024
Independent reflections	2949 [R_{int} = 0.2103, R_{sigma} = 0.1817]	14525 [R_{int} = 0.3698, R_{sigma} = 0.7771]
Data/restraints/parameters	2949/0/201	14525/87/409
Goodness-of-fit on F^2	0.880	0.703
Final R indexes [I>=2σ (I)]	R_1 = 0.0703, wR_2 = 0.1429	R_1 = 0.1010, wR_2 = 0.1906
Final R indexes [all data]	R_1 = 0.1386, wR_2 = 0.1659	R_1 = 0.3947, wR_2 = 0.2874
Largest diff. peak/hole / e Å^{-3}	1.22/-0.86	0.96/-0.95
Diffractometer	STOE IPDS T2	STOE IPDS T2
Remarks	-	A solvent mask was calculated and 15 electrons were found in a volume of 330 A^3 in 2 voids per unit cell. This is consistent with the presence of 0.375 CH_2Cl_2 per formula unit which account for 63 electrons per unit cell.
CCDC access code	2370203	2370204

Table S1.1: Crystallographic data and data collection parameters (continued)

	$[Tc(NS)Cl(pz^{HMe2})_4]\{Cl(pz^{H})_4\}$ (**5**$\{Cl(pz^{HMe2})_4\}$ · $(OPPh_3)$	$[Re(NS)Cl_2(PPh_3)_2(pz^{H})]$ (**6a**) · $0.5(CH_2Cl_2)$
Empirical formula	$C_{58}H_{79}Cl_2N_{17}OPSTc$	$C_{39.5}H_{35}Cl_3N_3P_2SRe$
Formula weight	1262.31	938.25
Temperature/K	240(2)	240(2)
Crystal system	Triclinic	Monoclinic
Space group	P-1	$P2_1/n$
a/Å	13.8262(8)	14.3065(8)
b/Å	13.8488(8)	14.0034(5)
c/Å	18.161(1)	19.759(1)
α/°	89.534(6)	90
β/°	70.525(5)	94.339(4)
γ/°	87.403(4)	90
Volume/Å^3	3275.0(4)	3948.2(3)
Z	2	4
ρ_{calc} / gcm^{-3}	1.280	1.579
μ / mm^{-1}	0.409	3.448
F(000)	1324.0	1860.0
Crystal size / mm^3	0.28 × 0.17 × 0.02	0.30 × 0.15 × 0.06
Radiation	MoKα (λ = 0.71073)	MoKα (λ = 0.71073)
2Θ range for data collection/°	6.718 to 58	9.372 to 52
Index ranges	$-18 \le h \le 18$, $-18 \le k \le 19$, $-24 \le l \le 24$	$-17 \le h \le 17$, $-17 \le k \le 17$, $-24 \le l \le 23$
Reflections collected	37304	22949
Independent reflections	17549 [R_{int} = 0.0869, R_{sigma} = 0.0826]	7694 [R_{int} = 0.0883, R_{sigma} = 0.0613]
Data/restraints/parameters	17549/0/747	7694/17/473
Goodness-of-fit on F^2	0.925	0.993
Final R indexes [I>=2σ (I)]	R_1 = 0.0493, wR_2 = 0.1226	R_1 = 0.0439, wR_2 = 0.1189
Final R indexes [all data]	R_1 = 0.0684, wR_2 = 0.1288	R_1 = 0.0594, wR_2 = 0.1273
Largest diff. peak/hole / e Å^{-3}	1.08/-1.72	1.62/-1.76
Diffractometer	STOE IPDS	STOE IPDS
Remarks		
CCDC access code	2370205	2370206

Table S1.1: Crystallographic data and data collection parameters (continued)

	$[Re(NS)Cl_2(PPh_3)_2(pz^{HMe2})]$ (**6b**)	$[Re(NS)Cl_2(PPh_3)_2(pz^{HPh})]$ (**6c**)
Empirical formula	$C_{41}H_{38}Cl_2N_3P_2ReS$	$C_{45}H_{38}Cl_2N_3P_2ReS$
Formula weight	923.84	971.88
Temperature/K	240(2)	200(2)
Crystal system	Monoclinic	Monoclinic
Space group	C2/c	$P2_1/n$
a/Å	31.571(2)	13.931(2)
b/Å	15.2737(8)	18.490(2)
c/Å	20.732(2)	15.755(2)
α/°	90	90
β/°	123.211(9)	96.597(9)
γ/°	90	90
Volume/Å^3	8364(1)	4031.2(8)
Z	8	4
ρ_{calc} / gcm^{-3}	1.467	1601
μ / mm^{-1}	3.191	3.315
F(000)	3680.0	1936.0
Crystal size / mm^3	0.30 × 0.25 × 0.17	0.30 x 0.02 x 0.02
Radiation	MoKα (λ = 0.71073)	MoKα (λ = 0.71073)
2Θ range for data collection/°	9.562 to 50	6.646 to 549.998
Index ranges	-37 ≤ h ≤ 37, -18 ≤ k ≤ 18, -24 ≤ l ≤ 24	-12 ≤ h ≤ 16, -21 ≤ k ≤ 21, -18 ≤ l ≤ 18
Reflections collected	21789	18950
Independent reflections	7286 [R_{int} = 0.0923, R_{sigma} = 0.0834]	7045 [R_{int} = 0.2016, R_{sigma} = 0.3223]
Data/restraints/parameters	7286/0/423	7045/39/448
Goodness-of-fit on F^2	0.907	0.787
Final R indexes [I>=2σ (I)]	R_1 = 0.0378, wR_2 = 0.0868	R_1 = 0.0740, wR_2 = 0.1228
Final R indexes [all data]	R_1 = 0.0571, wR_2 = 0.0911	R_1 = 0.1974, wR_2 = 0.1555
Largest diff. peak/hole / e Å^{-3}	1.28/-1.06	1.94/-1.25
Diffractometer	STOE IPDS	STOE IPDS
Remarks	-	-
CCDC access code	2370207	2370208

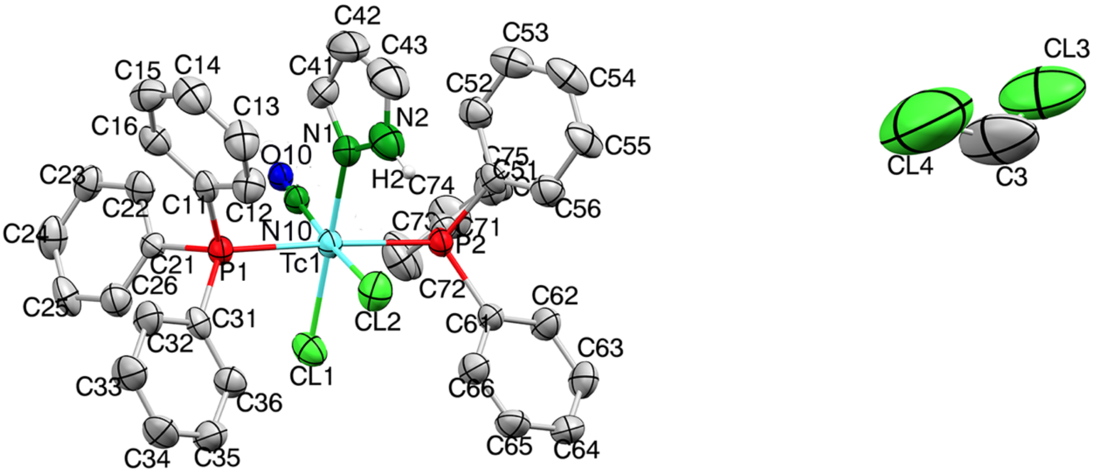

Figure S1.1. Ellipsoid representation of the structure of [Tc(NO)Cl$_2$(PPh$_3$)$_2$(pz^H)] (**1**) · CH$_2$Cl$_2$. The thermal ellipsoids are set at a 50% probability level. Hydrogen atoms are omitted for clarity.

Table S1.2. Selected bond lengths (Å) and angles (°) in [Tc(NO)Cl$_2$(PPh$_3$)$_2$(pz^H)] (**1**).

Tc1–N10	1.816(4)	Tc1–Cl1	2.447(1)	Tc1–Cl2	2.443(2)
Tc1–P1	2.474(2)	Tc1–P2	2.473(1)	Tc–N1	2.149(4)
N10–O10	0.946(4)				
N10–Tc1–Cl1	91.8(2)	N10–Tc1–Cl2	173.9(2)	N10–Tc1–P1	92.2(2)
N10–Tc1–P2	90.7(2)	N10–Tc1–N1	88.8(2)	Cl1–Tc1–Cl2	94.31(6)
Cl1–Tc1–P1	89.42(5)	Cl1–Tc1–P2	8915(5)	Cl1–Tc1–N1	179.2(2)
Cl2–Tc1–P1	87.47(6)	Cl2–Tc1–P2	89.73(6)	Cl2–Tc1–N1	85.1(2)
P1–Tc1–P2	176.75(6)	P1–Tc1–N1	90.0(1)	P2–Tc1–N1	91.4(1)
Tc1–N10–O10	178.4(5)				

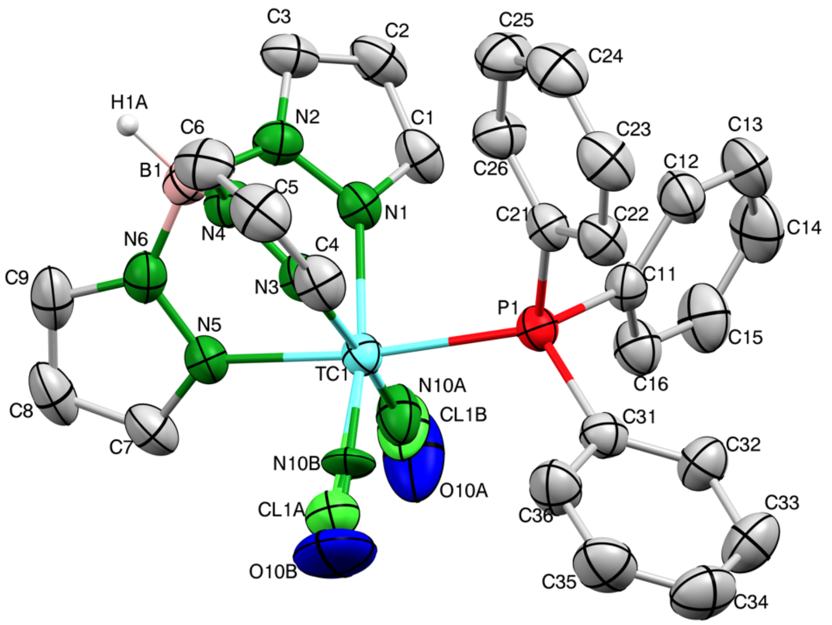

Figure S1.2. Ellipsoid representation of the molecular structure of [Tc(NO)Cl(PPh$_3$){HB(pz)$_3$}] (**2**) including the positional disorder between the Tc-Cl and Tc–N–O bonds. The thermal ellipsoids are set at a 50% probability level. Hydrogen atoms are omitted for clarity.

Table S1.3. Selected bond lengths (Å) and angles (°) in [Tc(NO)Cl(PPh$_3$){HB(pz)$_3$}] (**2**).

Tc1–N10	1.764(7)	Tc1–P1	2.4417(5)	Tc1–Cl1	2.425(2)
Tc1–N1	2.152(2)	Tc1–N3	2.172(1)	Tc1–N5	2.135(2)
N10–O10	1.13(1)				
N10–Tc1–P1	94.0(1)	N10–Tc1–Cl1	93.2(1)	N10–Tc1–N1	92.7(1)
N10–Tc1–N3	173.9(1)	N10–Tc1–N5	89.6(1)	P1–Tc1–Cl1	97.34(3)
P1–Tc1–N1	91.08(4)	P1–Tc1–N3	91.67(4)	P1–Tc1–N5	173.55(4)
Cl1–Tc1–N1	169.31(5)	Cl1–Tc1–N3	88.18(5)	Cl1–Tc1–N5	87.74(5)
N1–Tc1–N3	84.99(6)	N1–Tc1–N5	83.44(6)	N3–Tc1–N5	84.50(6)
Tc1–N10–O10	174.3(5)				

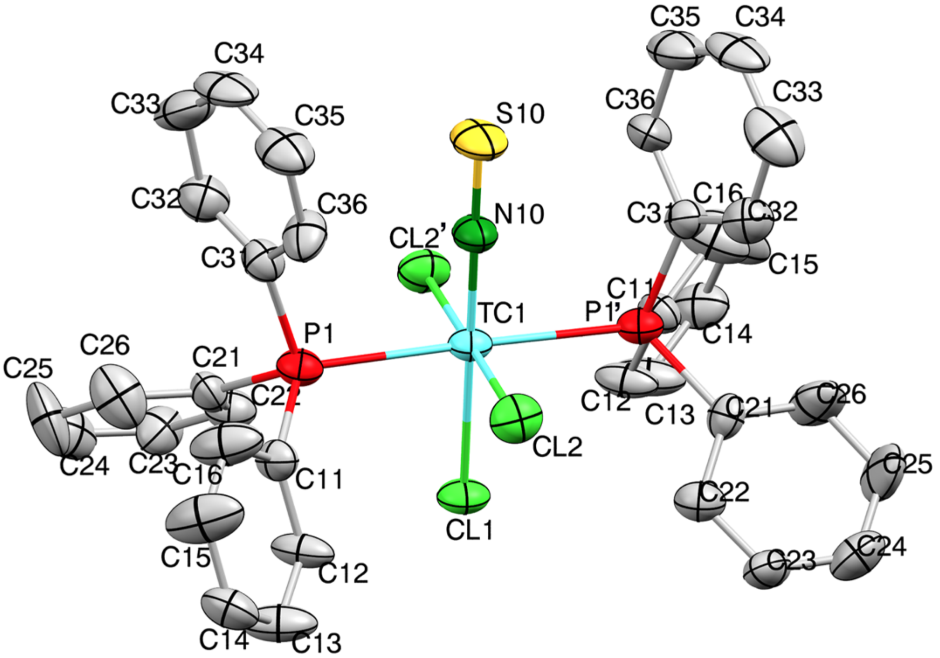

Figure S1.3. Ellipsoid representation of $[Tc(NS)Cl_3(PPh_3)_2]$ (**3**). The thermal ellipsoids are set at a 50% probability level. Hydrogen atoms are omitted for clarity.

Table S1.4. Selected bond lengths (Å) and angles (°) in $[Tc(NS)Cl_3(PPh_3)_2]$ (**3**).

Tc1–N10	1.78(1)	Tc1–Cl1	2.443(3)	Tc1–Cl2	2.357(2)
Tc1–P1	2.570(2)	N10–S10	1.51(1)		
N10–Tc1–Cl1	180	N10–Tc1–Cl2	90.27(2)	N10–Tc1–P1	90.87(7)
Cl1–Tc1–Cl2	89.73(7)	Cl1–Tc1–P1	89.13(7)	Cl2–Tc1–P1	92.77(8)
Cl2–Tc1–Cl2′	179.5(2)	P1–Tc1–P1′	178.3(1)	Tc1–N10–S10	180

‘ 1-x,y,1.5-z

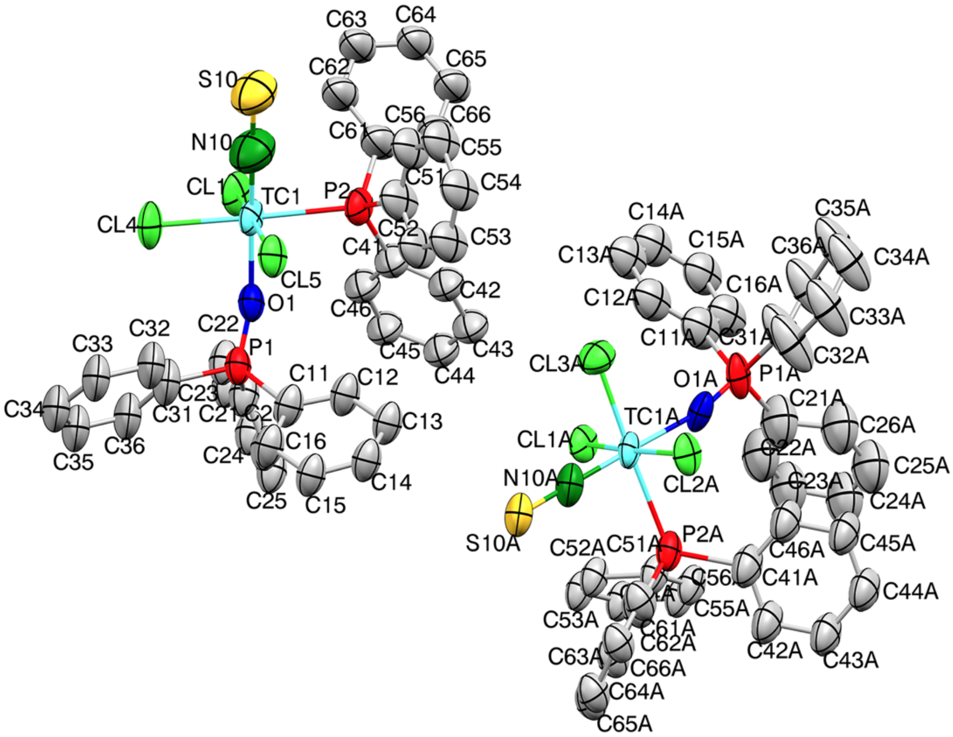

Figure S1.4. Ellipsoid representation of [Tc(NS)Cl3(PPh3)(OPPh3)] (**4**) including the positional disorder between the Re–N–S and Re–Cl bonds. The thermal ellipsoids are set at a 50% probability level. Hydrogen atoms are omitted for clarity.

Table S1.5. Selected bond lengths (Å) and angles (°) in [Tc(NS)Cl3(PPh3)(OPPh3)] (**4**).

Tc1–N10	1.68(2)	Tc1–Cl1	2.346(6)	Tc1–Cl2	2.356(6)
Tc1A–N10A	1.89(2)	Tc1A–Cl1A	2.333(6)	Tc1A–Cl2A	2.378(5)
Tc1–Cl3	2.368(6)	Tc1–O1	2.09(1)	Tc1–P2	2.552(7)
Tc1A–Cl3A	2.355(6)	Tc1A–O1A	2.13(1)	Tc1A–P2A	2.577(6)
N10–S10	1.55(2)	P1–O1	1.50(1)		
N10A–S10A	1.46(2)	P1A–O1A	1.48(1)		
N10–Tc1–Cl1	100.3(9)	N10–Tc1–Cl2	88.8(9)	N10–Tc1–Cl3	91.8(9)
N10A–Tc1A–Cl1A	95.0(6)	N10A–Tc1A–Cl2A	91.9(6)	N10A–Tc1A–Cl3A	92.5(6)
N10–Tc1–O1	175(1)	N10–Tc1–P2	89.0(9)	Cl1–Tc1–Cl2	170.1(2)
N10A–Tc1A–O1A	178.1(8)	N10A–Tc1A–P2A	88.6(6)	Cl1A–Tc1A–Cl2A	172.9(2)
Cl1–Tc1–Cl3	89.9(2)	Cl1–Tc1–O1	84.8(4)	Cl1–Tc1–P2	85.1(2)
Cl1A–Tc1A–Cl3A	91.0(2)	Cl1A–Tc1A–O1A	84.0(4)	Cl1A–Tc1A–P2A	90.9(2)
Cl2–Tc1–Cl3	93.7(2)	Cl2–Tc1–O1	86.1(4)	Cl2–Tc1–P2	91.2(2)
Cl2A–Tc1A–Cl3A	90.5(2)	Cl2A–Tc1A–O1A	89.0(4)	Cl2A–Tc1A–P2A	87.5(2)
Cl3–Tc1–O1	88.1(4)	Cl3–Tc1–P2	175.0(2)	O1–Tc1–P2	91.5(4)
Cl3A–Tc1A–O1A	89.3(4)	Cl3A–Tc1A–P2A	177.7(2)	O1A–Tc1A–P2A	89.7(4)
Tc1–N10–S10	174(2)	Tc1–O1–P1	154.4(9)		
Tc1A–N10A–S10A	174(1)	Tc1A–O1A–P1A	166.3(8)		

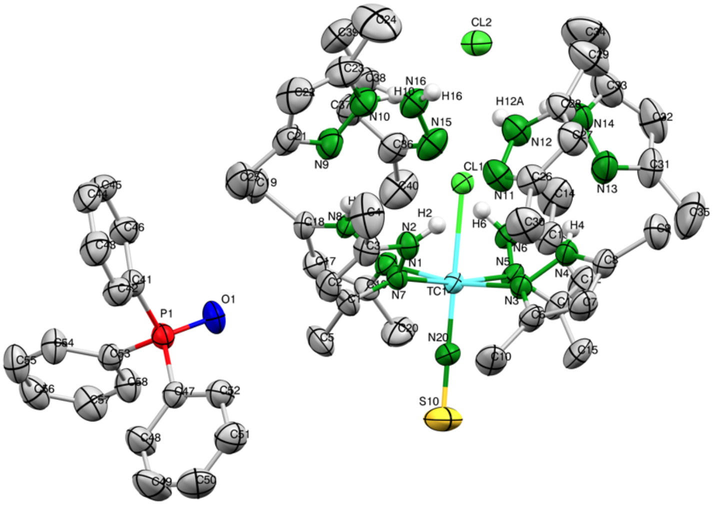

Figure S1.5. Ellipsoid representation of [Tc(NS)Cl(pz^{HMe2})$_4$]{Cl(pz^{HMe2})$_4$} (**5**{Cl(pz^{HMe2})$_4$}) · ($OPPh_3$). The thermal ellipsoids are set at a 50% probability level.

Table S1.6. Selected bond lengths (Å) and angles (°) in [Tc(NS)Cl(pz^{HMe2})$_4$]{Cl(pz^{HMe2})$_4$} (**5**{Cl(pz^{HMe2})$_4$}) · ($OPPh_3$).

Tc1–N20	1.733(2)	Tc–Cl1	2.4252(6)	Tc–N1	2.134(2)
Tc–N3	2.135(2)	Tc–N5	2.147(2)	Tc–N7	2.137(2)
N20–S10	1.572(2)	P1–O1	1.485(2)		
N20–Tc1–Cl1	179.66(8)	N20–Tc1–N1	94.05(9)	N20–Tc1–N3	94.58(9)
N20–Tc1–N5	94.12(9)	N20–Tc1–N7	95.49(9)	Cl1–Tc1–N1	86.29(5)
Cl1–Tc1–N3	85.46(6)	Cl1–Tc1–N5	85.54(6)	Cl1–Tc1–N7	84.48(5)
N1–Tc1–N3	89.68(8)	N1–Tc1–N5	171.82(7)	N1–Tc1–N7	87.94(8)
N3–Tc1–N5	89.21(8)	N3–Tc1–N7	169.79(7)	N5–Tc1–N7	91.74(8)
Tc1–N20–S10	179.4(2)				

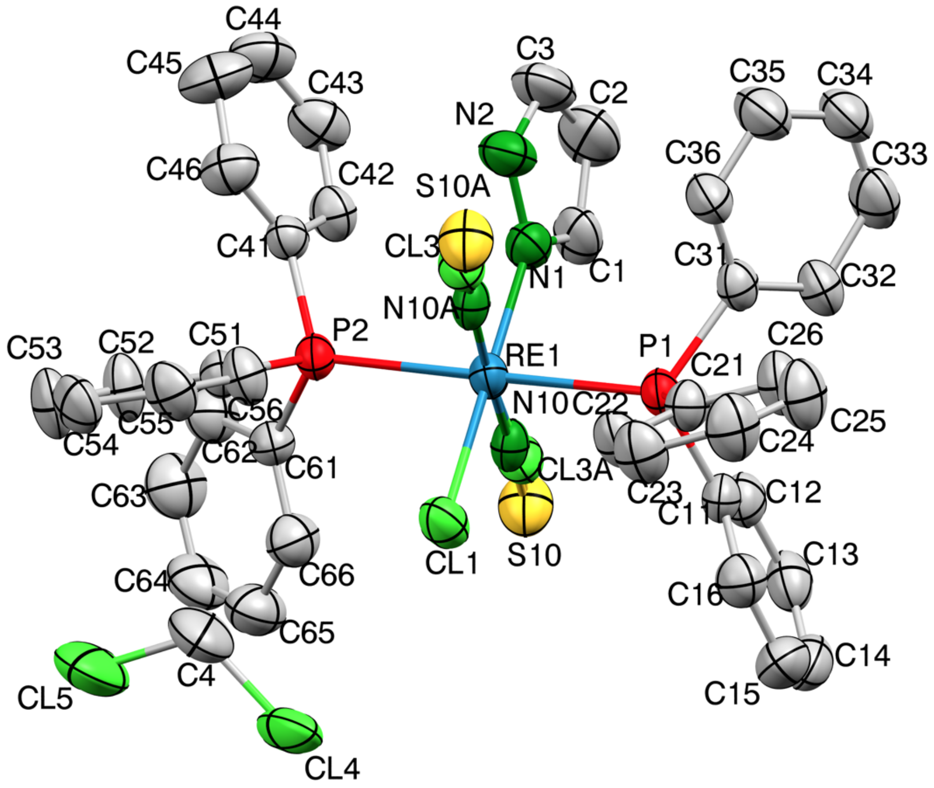

Figure S1.6. Ellipsoid representation of [Re(NS)Cl_2(PPh_3)$_2$(pz^H)] (**6a**) · 0.5(CH_2Cl_2) including the positional disorder for the NO and Cl3 ligands of the central molecular axis. The thermal ellipsoids are set at a 50% probability level.

Table S1.7. Selected bond lengths (Å) and angles (°) in [Re(NS)Cl_2(PPh_3)$_2$(pz^H)] (**6a**) · 0.5(CH_2Cl_2)

Re1–N10	1.738(8)	Re1–Cl1	2.438(2)	Re1–Cl3	2.488(4)
Re1–P1	2.476(2)	Re1–P2	2.470(2)	Re1–N1	2.142(5)
N10–S10	1.558(7)				
N10–Re1–Cl1	91.9(2)	N10–Re1–Cl3	174.8(2)	N10–Re1–P1	92.9(2)
N10–Re1–P2	90.9(2)	N10–Re1–N1	90.8(2)	Cl1–Re1–Cl3	93.3(1)
Cl1–Re1–P1	90.48(8)	Cl1–Re1–P2	88.69(8)	Cl1–Re1–N1	177.3(2)
Cl3–Re1–P1	87.51(9)	Cl3–Re1–P2	88.70(9)	Cl3–Re1–N1	84.1(2)
P1–Re1–P2	176.1(1)	P1–Re1–N1	88.8(2)	P2–Re1–N1	91.9(1)
Re1–N10–S10	178.3(3)				

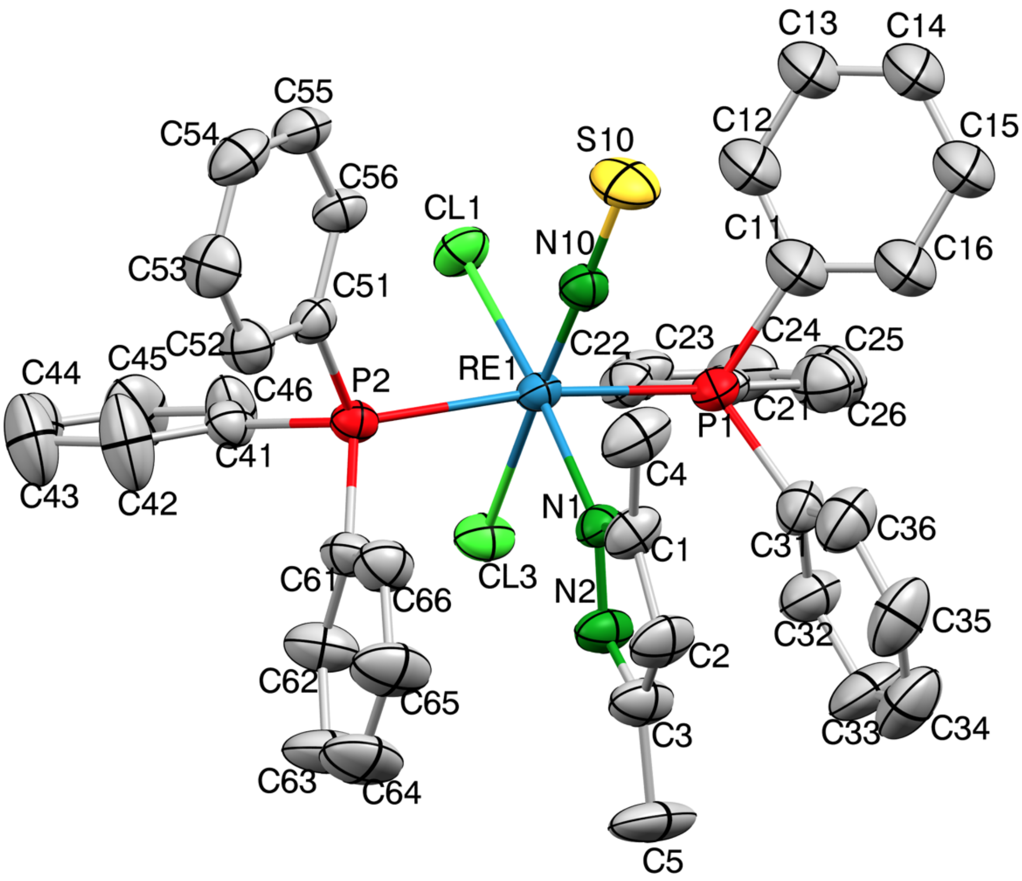

Figure S1.7. Ellipsoid representation of [Re(NS)Cl_2(PPh_3)$_2$(pz^{HMe2})] (**6b**). The thermal ellipsoids are set at a 50% probability level.

Table S1.8. Selected bond lengths (Å) and angles (°) [Re(NS)Cl_2(PPh_3)$_2$(pz^{HMe2})] (**6b**)

Re1–N10	1.737(5)	Re1–Cl1	2.445(2)	Re1–Cl3	2.468(1)
Re1–P1	2.464(2)	Re1–P2	2.496(2)	Re1–N1	2.158(4)
N10–S10	1.578(5)				
N10–Re1–Cl1	88.9(2)	N10–Re1–Cl3	176.6(2)	N10–Re1–P1	91.1(2)
N10–Re1–P2	97.7(2)	N10–Re1–N1	95.8(2)	Cl1–Re1–Cl3	90.25(5)
Cl1–Re1–P1	91.49(5)	Cl1–Re1–P2	90.44(5)	Cl1–Re1–N1	175.0(1)
Cl3–Re1–P1	85.61(6)	Cl3–Re1–P2	85.60(5)	Cl3–Re1–N1	85.1(1)
P1–Re1–P2	171.01(5)	P1–Re1–N1	89.9(1)	P2–Re1–N1	87.4(1)
Re1–N10–S10	173.0(3)				

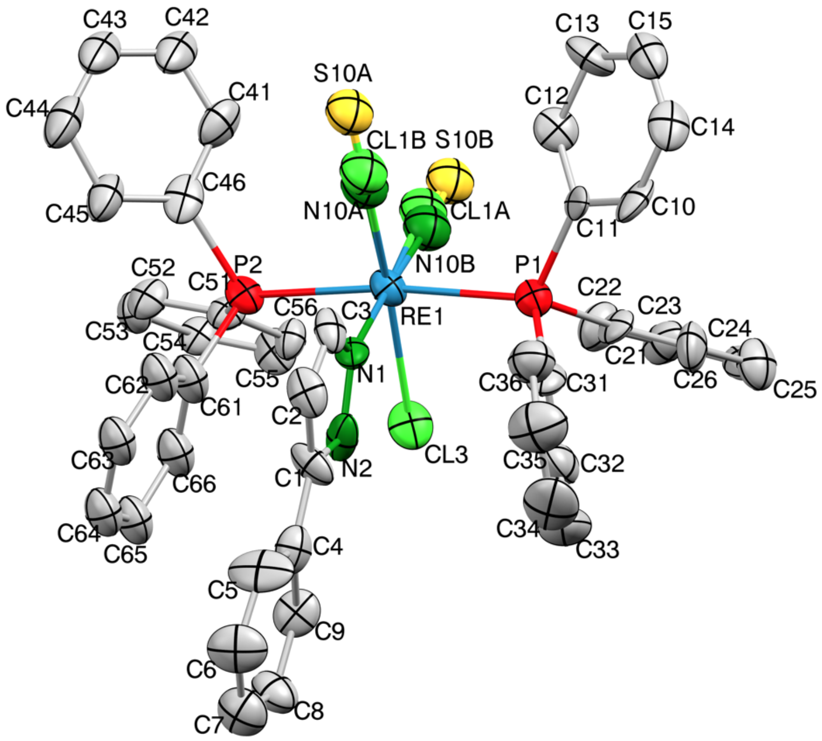

Figure S1.8. Ellipsoid representation of [Re(NS)Cl_2(PPh_3)$_2$(pz^{HPh})] (**6c**) including the positional disorder between the NS and the Cl1 ligands. The thermal ellipsoids are set at a 50% probability level

Table S1.9. Selected bond lengths (Å) and angles (°) in [Re(NS)Cl_2(PPh_3)$_2$(pz^{HPh})] (**6c**)

Re1–N10A	1.98(2)	Re1–Cl1A	2.441(8)	Re1–Cl3	2.449(4)
Re1–P1	2.477(4)	Re1–P2	2.468(5)	Re1–N1	2.18(2)
N10–S10	1.36(2)				
N10A–Re1–Cl1A	93(1)	N10A–Re1–Cl3	171(1)	N10A–Re1–P1	94.4(9)
N10A–Re1–P2	92.0(9)	N10A–Re1–N1	85(1)	Cl1A–Re1–Cl3	95.6(2)
Cl1A–Re1–P1	94.2(2)	Cl1A–Re1–P2	88.0(2)	Cl1A–Re1–N1	177.6(4)
Cl3–Re1–P1	85.4(2)	Cl3–Re1–P2	87.8(2)	Cl3–Re1–N1	85.8(4)
P1–Re1–P2	173.1(2)	P1–Re1–N1	87.9(4)	P2–Re1–N1	90.1(4)
Re1–N10–S10	173(2)				

2. Spectroscopic data

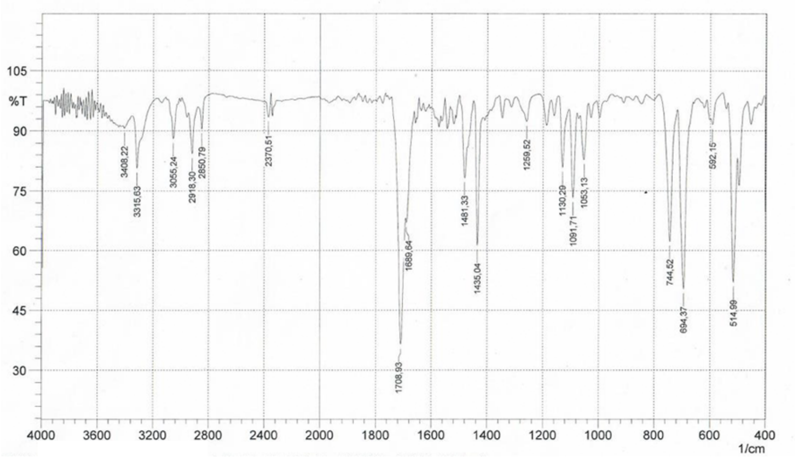

Figure S2.1: IR (KBr) spectrum of [Tc(NO)Cl$_2$(PPh$_3$)$_2$(pz^H)] (**1**)

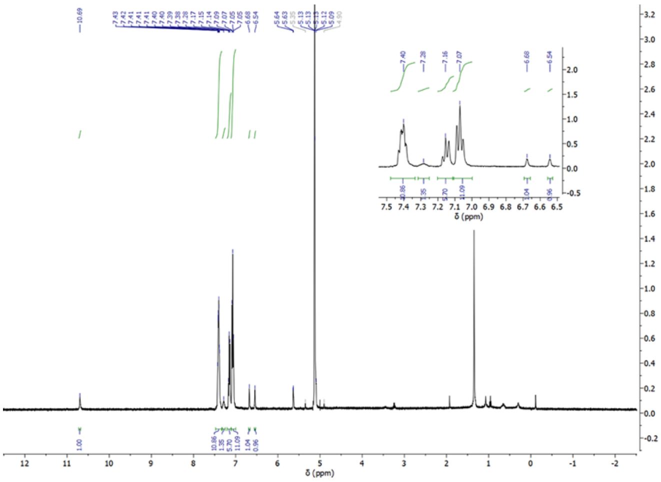

Figure S2.2: ^{1}H NMR spectrum of [Tc(NO)Cl$_2$(PPh$_3$)$_2$(pz^H)] (**1**) in CDCl$_3$.

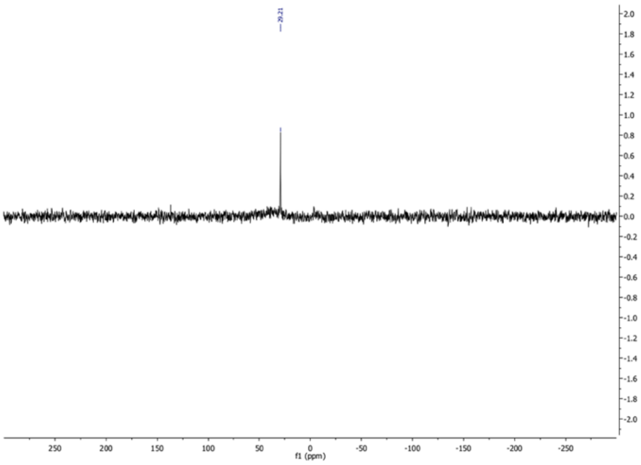

Figure S2.3: ^{31}P NMR spectrum of [Tc(NO)Cl$_2$(PPh$_3$)$_2$(pz^H)] (**1**) in CDCl$_3$.

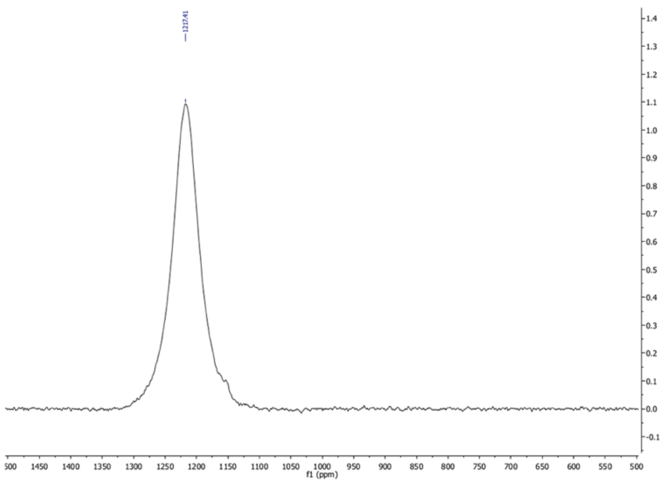

Figure S2.3: ^{99}Tc NMR spectrum of [Tc(NO)Cl$_2$(PPh$_3$)$_2$(pz^H)] (**1**) in CDCl$_3$.

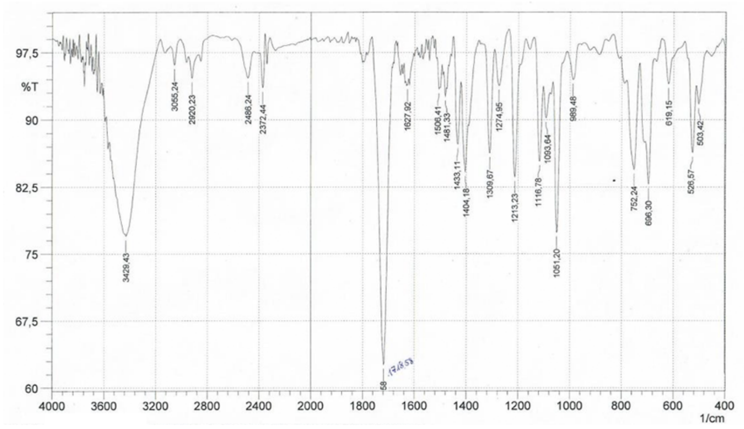

Figure S2.4: IR (KBr) spectrum of [Tc(NO)Cl(PPh$_3$){HB(pz)$_3$}] (**2**).

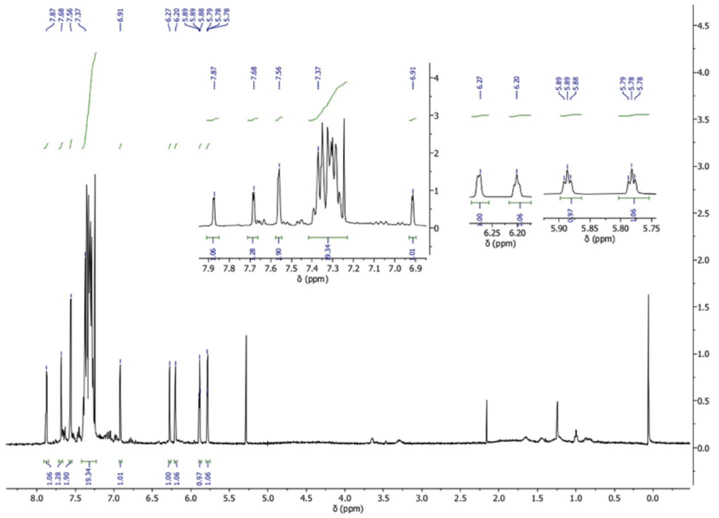

Figure S2.5: ^{1}H NMR spectrum of [Tc(NO)Cl(PPh$_3$){HB(pz)$_3$}] (**2**) in CDCl$_3$.

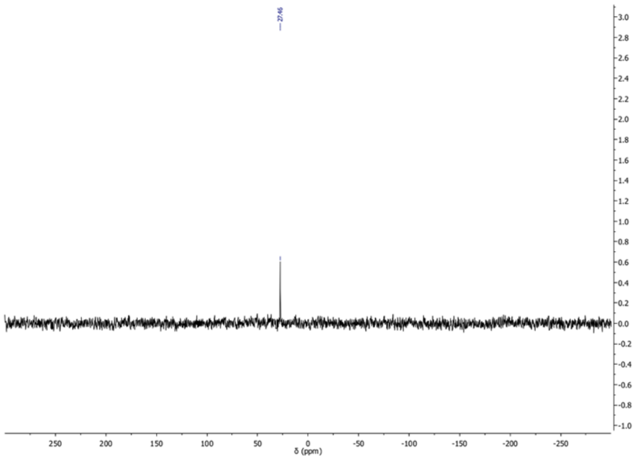

Figure S2.6: ^{31}P NMR spectrum of $[Tc(NO)Cl(PPh_3)\{HB(pz)_3\}]$ (**2**) in $CDCl_3$.

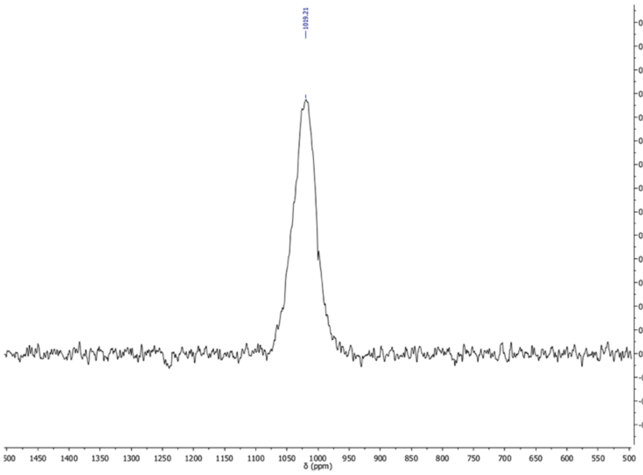

Figure S2.7: ^{99}Tc NMR spectrum of $[Tc(NO)Cl(PPh_3)\{HB(pz)_3\}]$ (**2**) in $CDCl_3$.

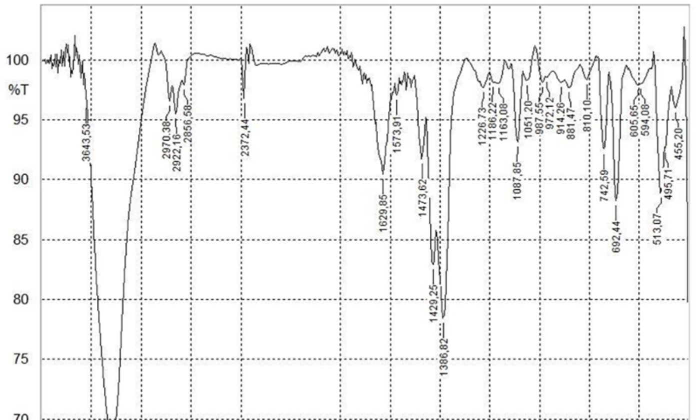

Figure S2.8: IR (KBr) spectrum of $[Tc(NS)Cl_3(PPh_3)_2]$ (**3**).

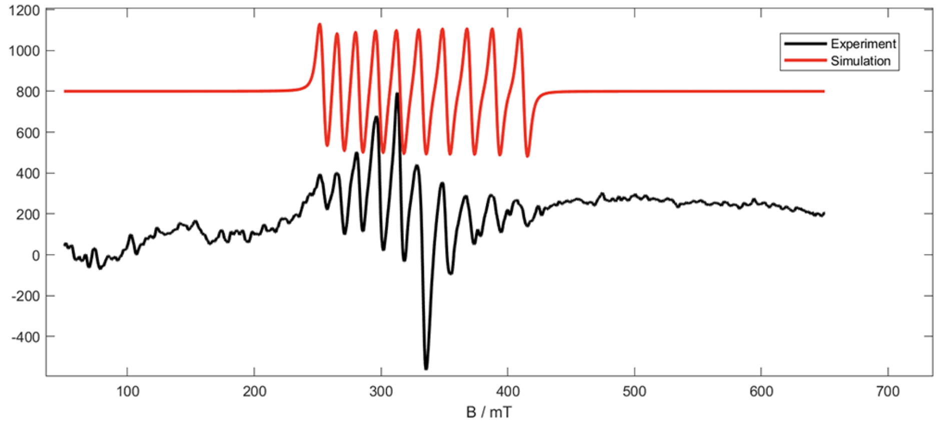

Figure S2.9: Solution EPR spectrum of $[Tc(NS)Cl_3(PPh_3)_2]$ (**3**) in CH_2Cl_2 at room-temperature.

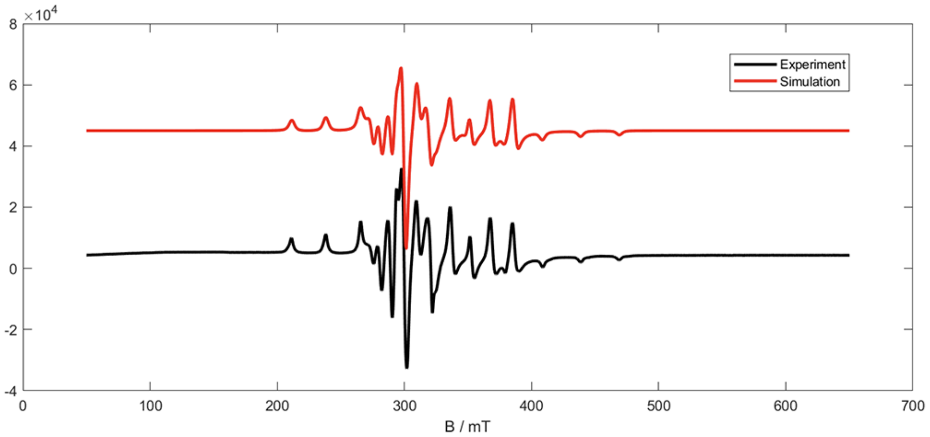

Figure S2.10: Solution EPR spectra of $[Tc(NS)Cl_3(PPh_3)_2]$ (**3**) in CH_2Cl_2 at T = 77 K.

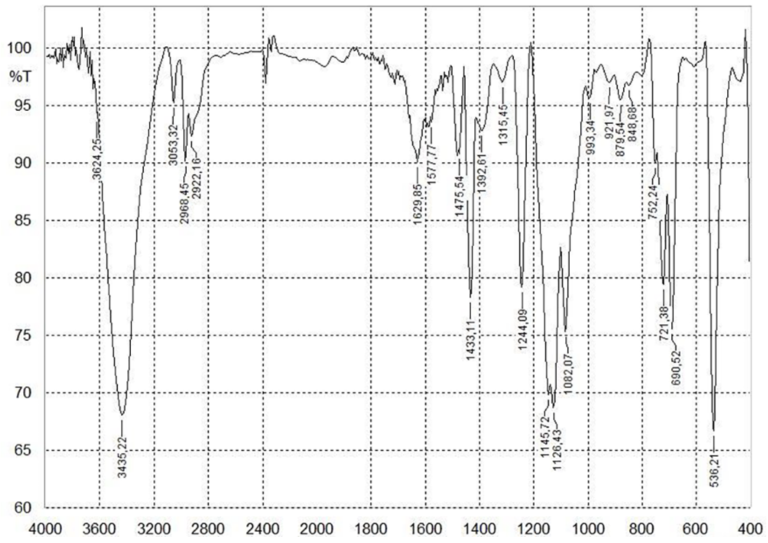

Figure S2.11: IR (KBr) spectrum of $[Tc(NS)Cl_3(PPh_3)(OPPh_3)]$ (**4**).

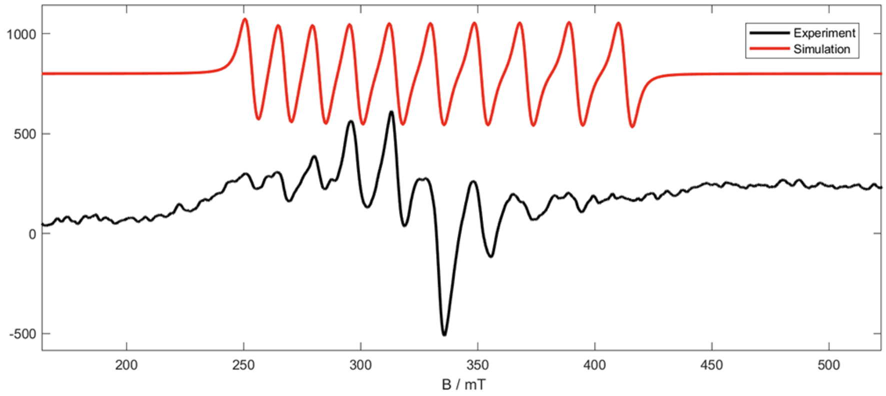

Figure S2.12: Solution EPR spectrum of $[Tc(NS)Cl_3(PPh_3)(OPPh_3)]$ (**4**) in CH_2Cl_2 at room-temperature.

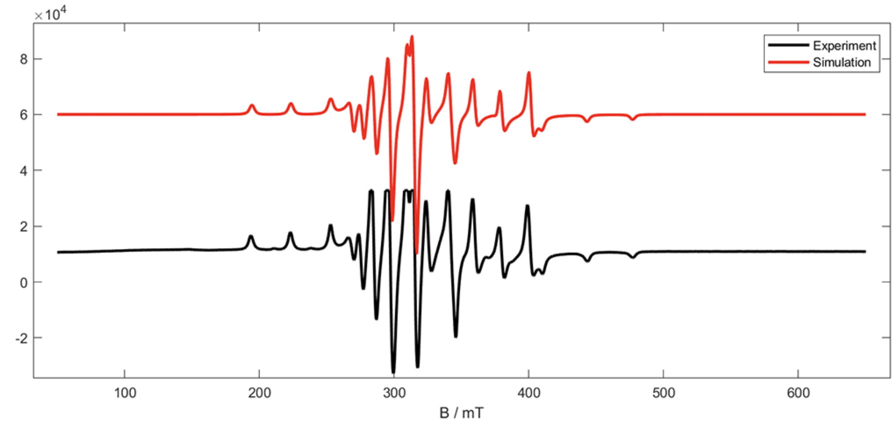

Figure S2.13: Solution EPR spectra of $[Tc(NS)Cl_3(PPh_3)(OPPh_3)]$ (**4**) in CH_2Cl_2 at T = 77 K.

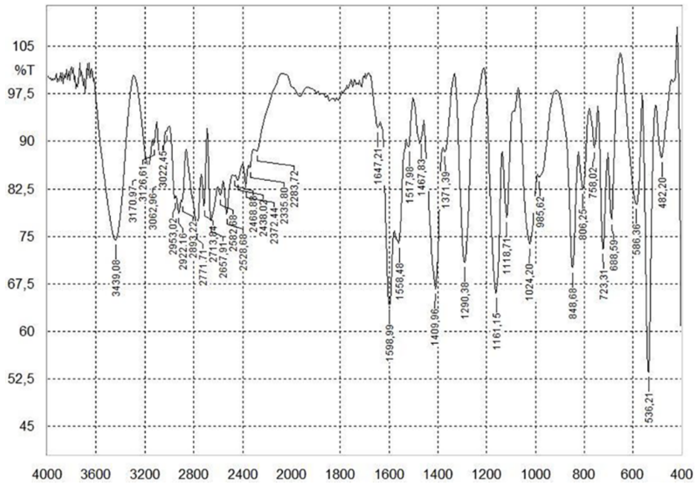

Figure S2.14: IR (KBr) spectrum of $[Tc(NS)Cl(pz^{HMe2})_4]\{Cl(pz^{HMe2})_4\}$ ($\mathbf{5}\{Cl(pz^{HMe2})_4\}$) · $(OPPh_3)$.

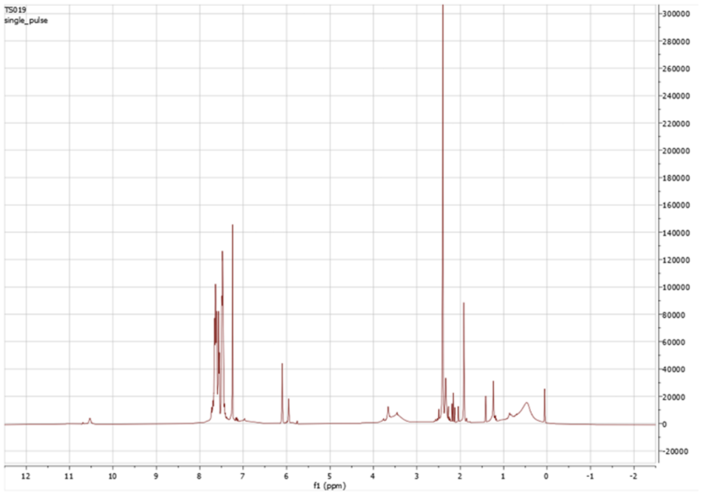

Figure S2.15: ^{1}H NMR spectrum of [Tc(NS)Cl(pz^{HMe2})$_4$]{Cl(pz^{HMe2})$_4$} (**5**{Cl(pz^{HMe2})$_4$}) · (OPPh$_3$) in CDCl$_3$.

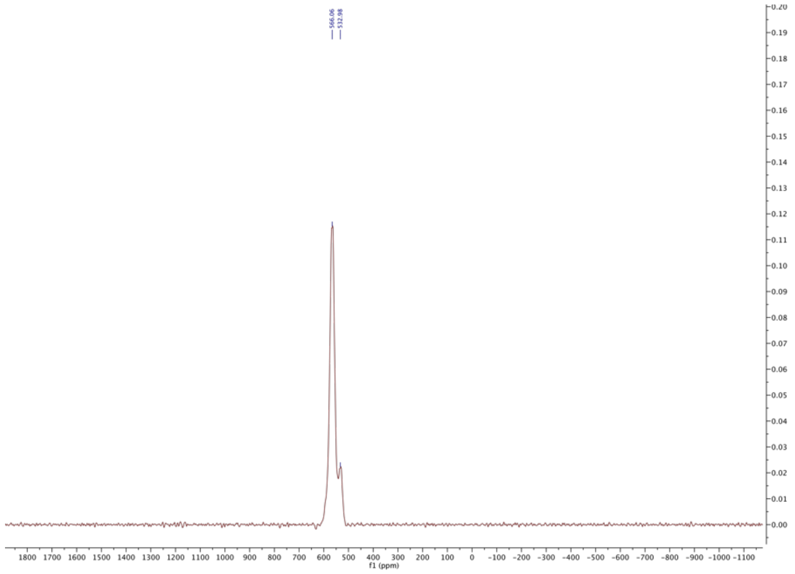

Figure S2.16: ^{99}Tc NMR spectrum of [Tc(NS)Cl(pz^{HMe2})$_4$]{Cl(pz^{HMe2})$_4$} (**5**{Cl(pz^{HMe2})$_4$}) · (OPPh$_3$) in CDCl$_3$.

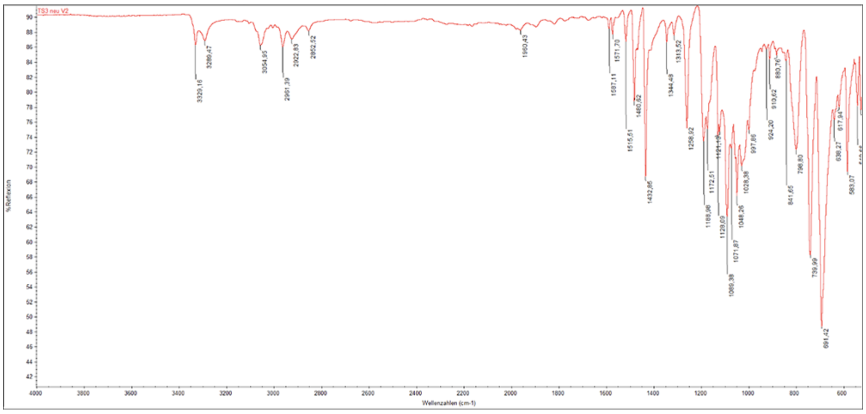

Figure S2.17: IR (ATR) spectrum of [Re(NS)Cl$_2$(PPh$_3$)$_2$(pz^{H})] (**6a**).

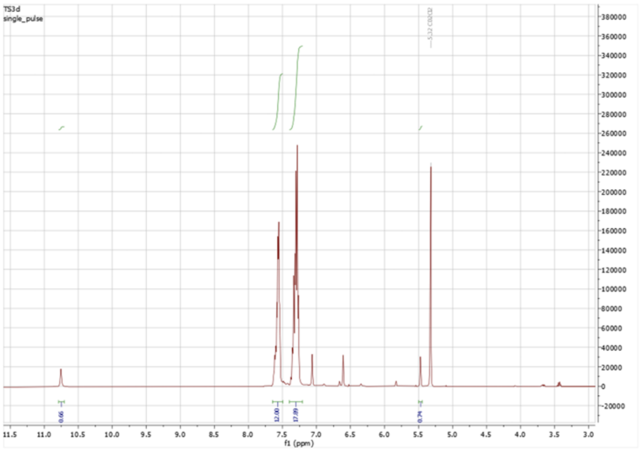

Figure S2.18: ^{1}H NMR spectrum of $[Re(NS)Cl_2(PPh_3)_2(pz^H)]$ (**6a**) in CD_2Cl_2.

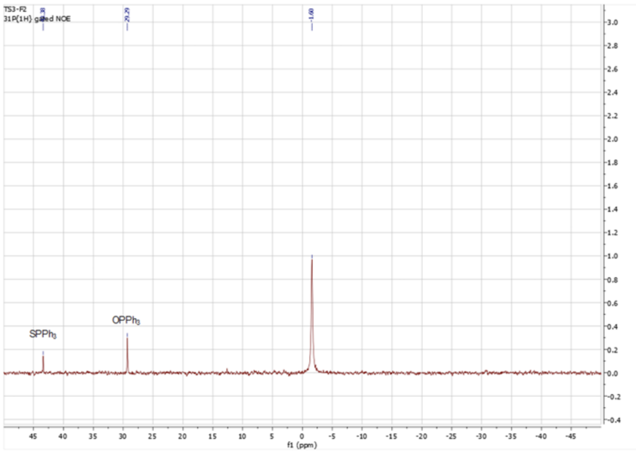

Figure S2.19: ^{31}P NMR spectrum of $[Re(NS)Cl_2(PPh_3)_2(pz^H)]$ (**6a**) in CD_2Cl_2.

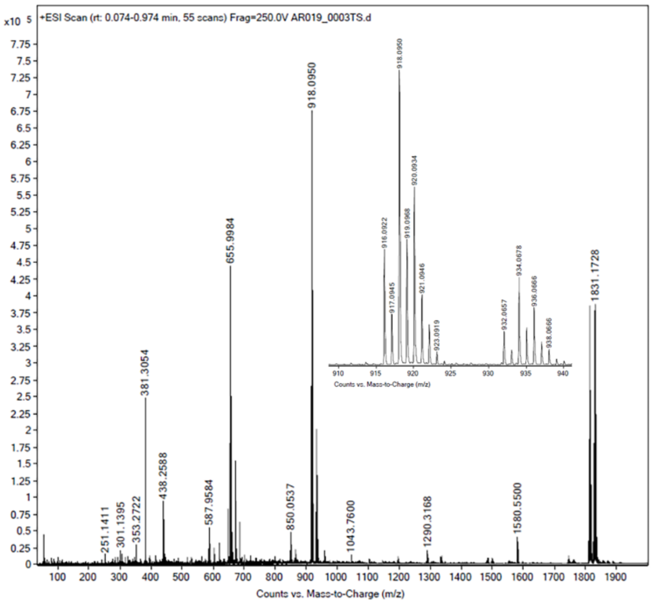

Figure S2.20: ESI+ mass spectrum of $[Re(NS)Cl_2(PPh_3)_2(pz^H)]$ (**6a**).

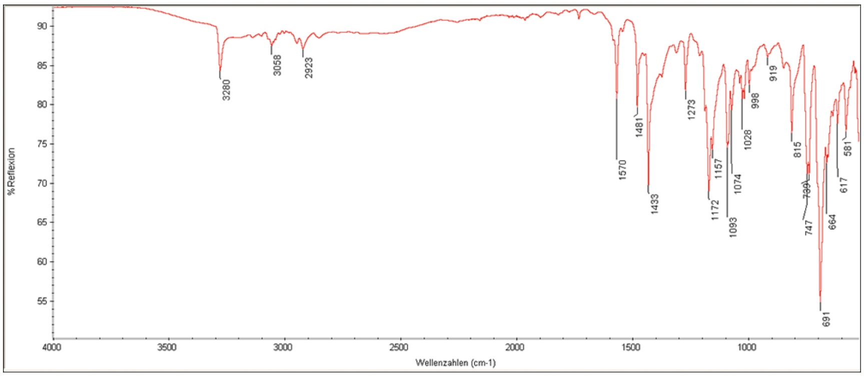

Figure S2.21: IR (ATR) spectrum of [Re(NS)Cl_2(PPh_3)$_2$(pz^{HMe2})] (**6b**).

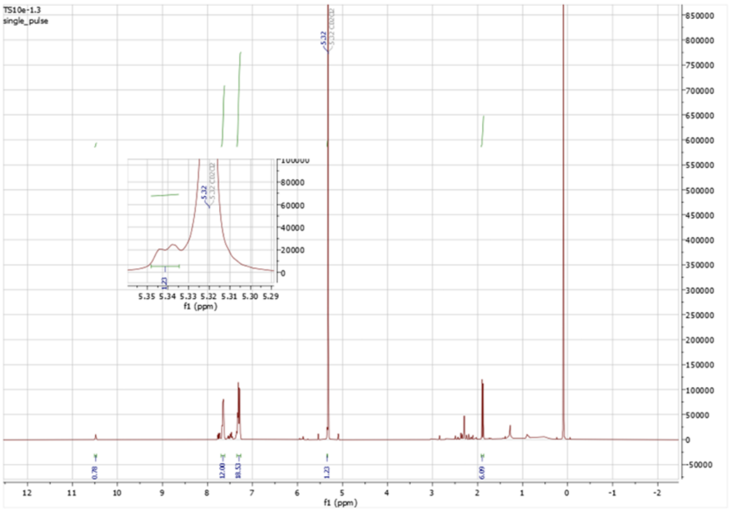

Figure S2.22: ^{1}H NMR spectrum of [Re(NS)Cl_2(PPh_3)$_2$(pz^{HMe2})] (**6b**) in CD_2Cl_2.

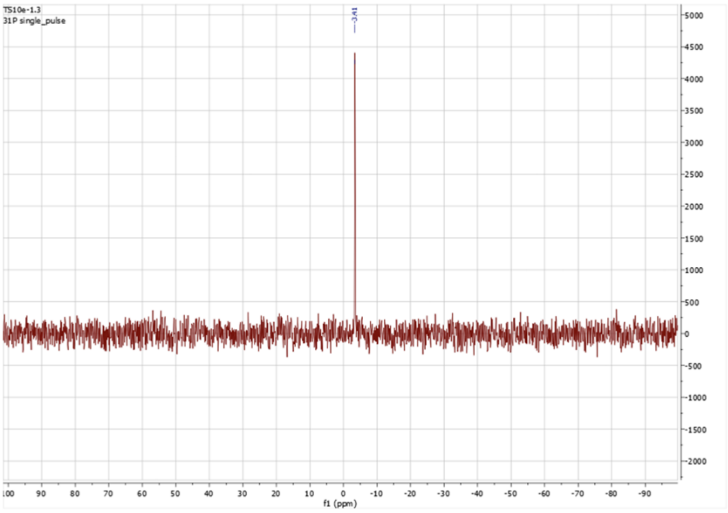

Figure S2.23: ^{31}P NMR spectrum of [Re(NS)Cl_2(PPh_3)$_2$(pz^{HMe2})] (**6b**) in CD_2Cl_2.

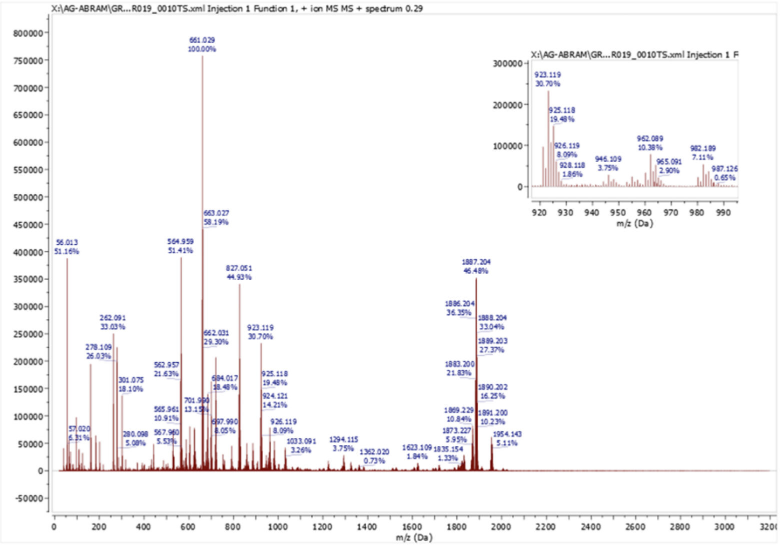

Figure S2.24: ESI+ mass spectrum of [Re(NS)Cl$_2$(PPh$_3$)$_2$(pz^{HMe2})] (**6b**).

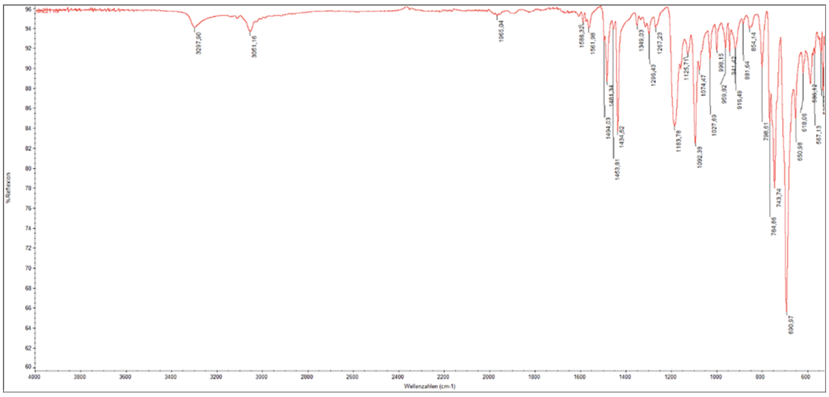

Figure S2.25: IR (ATR) spectrum of [Re(NS)Cl$_2$(PPh$_3$)$_2$(pz^{HPh})] (**6c**).

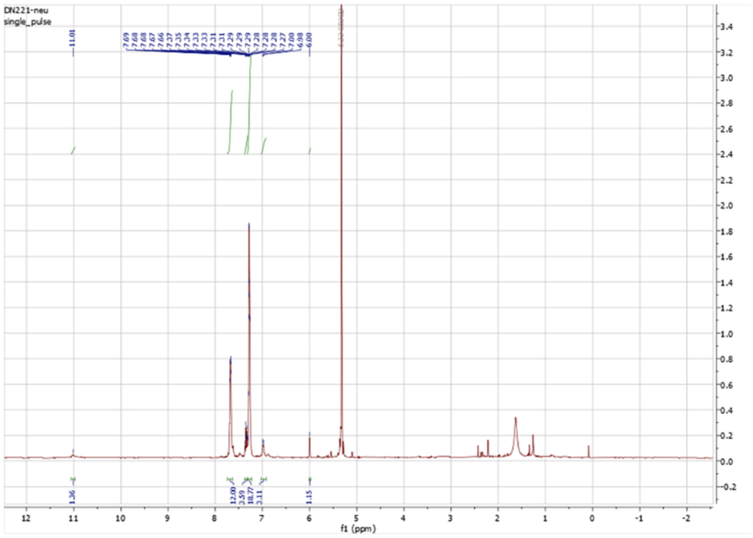

Figure S2.26: ^{1}H NMR spectrum of [Re(NS)Cl$_2$(PPh$_3$)$_2$(pz^{HPh})] (**6c**) in CD_2Cl_2.

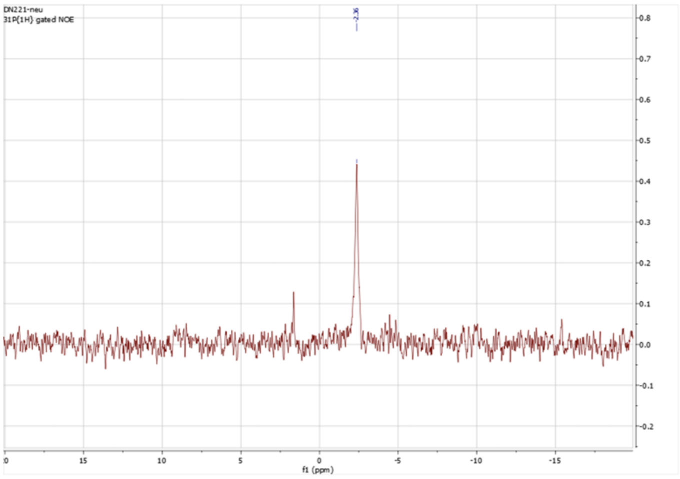

Figure S2.27: ^{31}P NMR spectrum of $[Re(NS)Cl_2(PPh_3)_2(pz^{HPh})]$ (**6c**) in CD_2Cl_2.

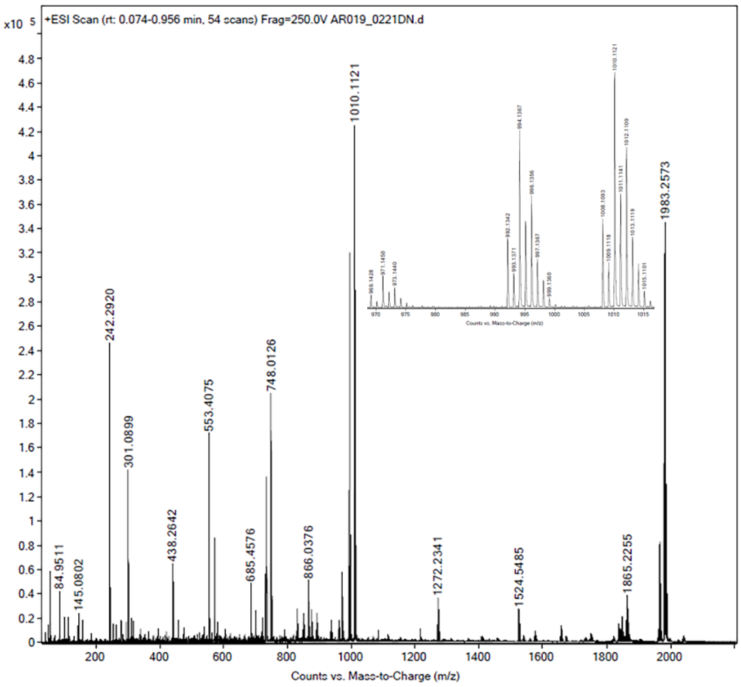

Figure S2.28: ESI+ mass spectrum of $[Re(NS)Cl_2(PPh_3)_2(pz^{HPh})]$ (**6c**).
